# Drug-induced self-assembled nanovesicles for doxorubicin resistance reversal via autophagy inhibition and delivery synchronism

**DOI:** 10.7150/thno.70852

**Published:** 2022-05-13

**Authors:** Juan Wang, Liyan Qiu

**Affiliations:** Ministry of Educational (MOE) Key Laboratory of Macromolecular Synthesis and Functionalization, Department of Polymer Science and Engineering, Zhejiang University, Hangzhou 310027, China

**Keywords:** multidrug resistance, autophagy, doxorubicin, chloroquine, nanovesicle

## Abstract

**Background:** As a classical autophagy inhibitor, CQ has been supposed to increase the sensitivity of tumors to chemotherapeutics. However, there exists a quite huge gap between laboratory research and clinical application, which is related to the distinct pharmacokinetic behavior of CQ to a great extent.

**Methods:** Based on amphiphilic copolymer PPAP, a pH-responsive drug-induced self-assembled nanovesicle, named DC-DIV/C, was constructed to load DOX⋅HCl and CQ. The physicochemical properties of DC-DIV/C were characterized. To validate the cooperative action and delivery synchronism of DOX⋅HCl and CQ, cytotoxicity, apoptosis, cellular uptake and autophagy assay were investigated in DOX⋅HCl resistant cancer cells. The pharmacokinetic character and antitumor effect of DC-DIV/C were evaluated on rats and nude mice bearing xenograft drug-resistant K562/ADR tumors, respectively.

**Results:** DC-DIV/C could simultaneously encapsulate DOX·HCl and CQ at the optimal ratio of 1:2. In vitro and in vivo tests confirmed that DC-DIV/C acted as an excellent vehicle for the synchronous delivery of DOX⋅HCl and CQ during the process of blood circulation, cellular uptake and intracellular release. Furthermore, CQ accomplished autophagy inhibition to reduce the IC_50_ of DOX⋅HCl resistant cancer cells. Consequently, DC-DIV/C exhibited the extremely improved anti-tumor effect with 84.52% TIR on K562/ADR tumor.

**Conclusion:** This study provides a promising and powerful strategy to achieve enhanced treatment outcomes for the precise combination therapy.

## Introduction

As a cytoprotective mechanism, autophagy can allow tumor cells to survive from chemotherapy, thereby mediating multidrug resistance (MDR) 1, 2. During this process, damaged organelles and subcellular debris are engulfed in double-membrane autophagosomes, and then these autophagosomes fuse with lysosomes to form autolysosomes to degrade those internalized substances under the action of lysosomal hydrolases, producing amino acids, fatty acids, sugars and nucleotides, which revert to the cytosol and re-enter the intracellular anabolic circuits 1, 3-6. Consequently, autophagy becomes an important energy source for tumor cell survival as the energy acquired for tumor growth is weakened by chemotherapy 7. The updated researches have confirmed that autophagy is closely related the development of MDR of chemotherapeutics in mutated cancers, such as melanoma, pediatric brain tumors and pancreatic adenocarcinoma 8, 9. Autophagy inhibition can disrupt tumor metabolism, resulting in multiple metabolic consequences including impaired mitochondrial metabolism, depletion of nucleotide pools, redox imbalance and reduced energy charge 10. Thereby, autophagy inhibition can facilitate the efficiency of chemotherapy in MDR tumor 11. Based on this mechanism, several potential autophagy inhibitors at diverse targets in the autophagy pathway have been increasingly concerned in preclinical mouse models. Among them, chloroquine (CQ) is quite a special member. CQ used to be a traditional anti-malaria drug, but it was initiated as a typical pharmacologic inhibitor of autophagy in 1998. Currently, CQ has been recognized as a classical autophagy inhibitor, which can inhibit autophagosome-lysosome fusion by elevating lysosomal pH and thereby can block the degradation of autophagic cargo, with the increased expression of autophagosome associated protein LC3-II and autophagy substrate protein p62 in tumor cells 12, 13. At present, CQ has been tried as sensitizer to chemotherapy or radiotherapy in a series of clinical trials approved by FDA 14, 15. It was reported that the median survival of the patients suffering glioblastoma was prolonged when treated with the combination of CQ and temozolomide 16. However, the overall clinical response has varied widely. The majority of initial studies hasn't announced outcome and others found no significant improvement in the treatments. Moreover, with the relatively high dosage of 400 mg daily - 600 mg twice daily via oral administration for CQ 8, the dose-limiting toxicities occurred 17, such as myelosuppression, which impedes the effective performance of cancer therapy. This disappointing information resulted in a quite huge gap of CQ sensibilization action between laboratory research and clinical application. As the analogue of CQ, hydroxychloroquine exhibits the similar bioactivity to CQ but with less toxicity, therefore more laboratory studies involving hydroxychloroquine as sensitizer have been carried out 18, 19. However, the treatment outcome in clinical trials is still not positively confirmed.

To elucidate this problem, we have to take an account of the distinct pharmacokinetic behavior of CQ. CQ is well absorbed into circulation system after oral taken and displays a high plasma protein binding rate with an extent of 60 % 20, 21. More specially, CQ has large apparent distribution volume up to 800 L/kg in the body since it can distribute extensively in various tissues, such as liver, lung, kidney, uveal tract, heart, brain, and so on 22, 23. In addition, most of the tissues showed much higher concentrations at 24 h than at 1 h after dosing and the accumulation is serious with a quite long half-life ranging from 20 days to 60 days 23. As we know, nanoparticle carrier is definitely an available kind of strategies to amend PK behavior of drug in body and accomplish targeted drug delivery towards cancer via the enhanced permeability and retention (EPR) effect 24-26. In our previous research, DOX·HCl and CQ at the optimized ratio were tried to be co-delivered by means of liposomes 27, 28. In that case, regretably, CQ released faster than DOX·HCl under normal physiological condition. Since DOX·HCl and CQ have totally different PK characters, when more CQ leakage happened, the synchronization action rhythm of CQ with DOX·HCl could not be guaranteed. Therefore, to completely exert the sensitizing effect of CQ for DOX·HCl, an ideal vehicle with ensured synchronism is necessary.

**Scheme 1:**
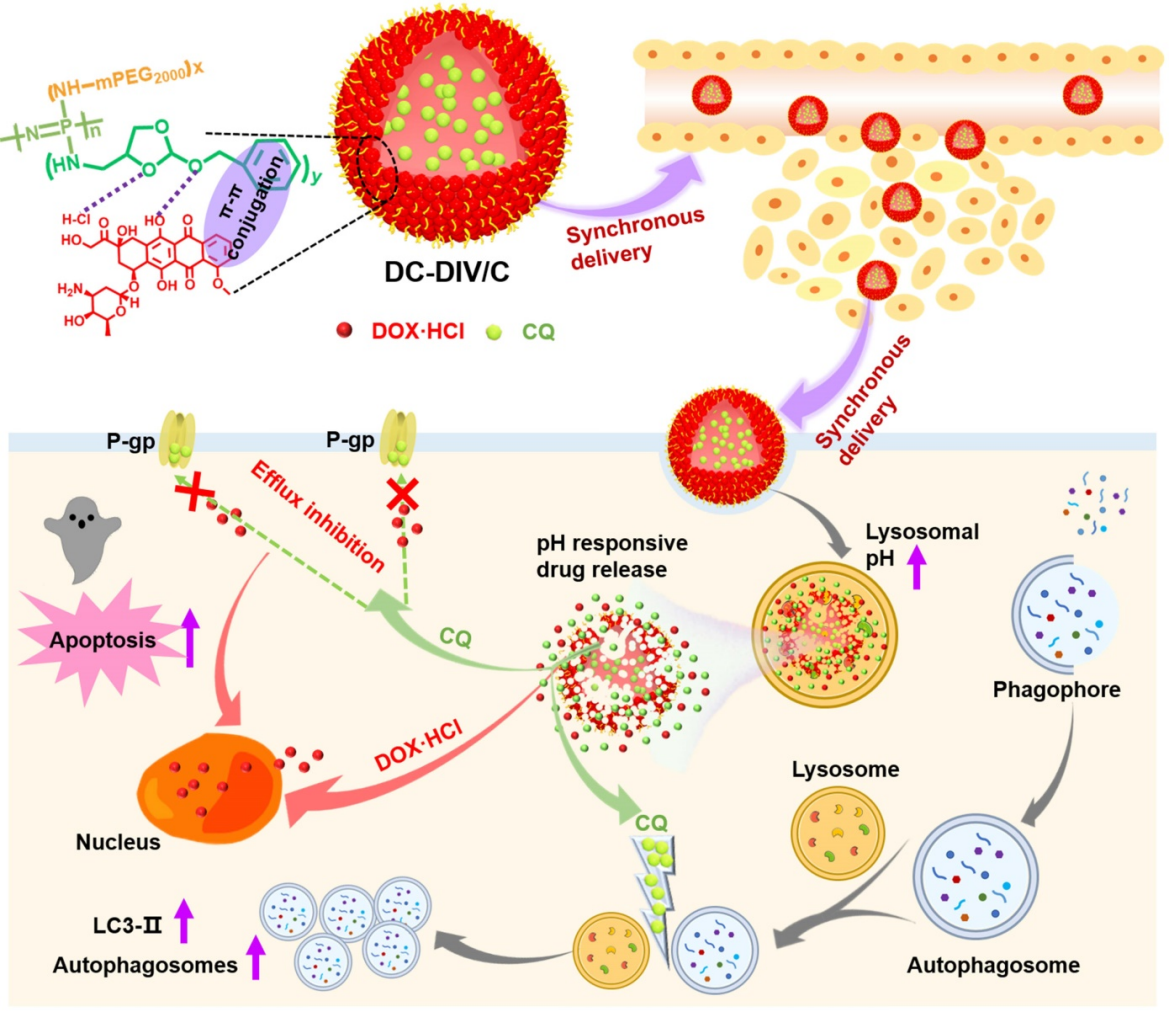
Structural composition and the nominated mechanism of DOX·HCl/CQ co-loaded drug-induced self-assembled nano-vesicles DC-DIV/C based on amphiphilic polymer PPAP for MDR treatment.

Herein, a pH-responsive CQ and DOX·HCl co-loaded nanovesicle (DC-DIV/C) was designed to achieve resistance reversal of DOX·HCl against tumor via synchronous delivery and synergistic effect (**Scheme 1**). In this delivery system, polyphosphazene derivate PPAP bearing (4-aminomethyl-2-benzyloxy-[1,3]-dioxolan) (ABD) with a five-membered cyclic ortho ester and a benzene ring was first synthesized. In virtue of the interaction between DOX·HCl and PPAP 29, drug-induced self-assembled nanovesicle with cholesteryl hemisuccinate-incorporation (DIV/C) was constructed and simultaneously encapsulated CQ to get DC-DIV/C, which displayed multiple advantages as follows. Firstly, DOX⋅HCl and CQ are both water soluble small molecules, which can be hardly encapsulated into nanoparticles unless chemical bonding method or active loading method is applied. However, in this work, the co-loading process via dialysis was very simple and the loading contents of these two drugs at the optimal ratio could reach high level since they can be respectively located in the membrane and center aqueous chamber of DIV. Secondly, due to the interaction between DOX·HCl and PPAP as well as the incorporation of cholesteryl hemisuccinate into the membrane of DIV, the premature leakage of DOX⋅HCl and CQ could be inhibited and their loading ratio could be maintained during blood circulation. Thirdly, DC-DIV/C would render CQ and DOX·HCl selectively reach tumor tissues via EPR effect and be internalized into tumor cells. Afterwards, the ortho esters in PPAP would be hydrolyzed by intracellular acid stimulation, triggering the dissociation of DIV/C and entire release of CQ and DOX·HCl 30. Therefore, DC-DIV/C is expected to realize the delivery synchronism of DOX⋅HCl and CQ at the optimal ratio during the in vivo process of blood circulation, tumor site accumulation, cellular uptake and intracellular release in tumor. Using doxorubicin-resistant cancer cells as models, a series of in vitro and in vivo experiments were performed to validate the enhanced anticancer effect DC-DIV/C and explore the cooperative action mechanism.

## Materials and Methods

### Materials

Pyridinium *p*-toluenesulfonate, 3-Amino-1,2-propanediol, *p*-toluenesulfonic acid monohydrate, and benzyl alcohol were acquired from Aladdin Ltd. (Shanghai, China). Ethyl trifluoroacetate was purchased from Adamas Reagent Co. Ltd. (Shanghai, China). Trimethyl orthoformate was purchased from Alfa Aesar (Shanghai, China). Cholesteryl hemisuccinate, *p*-nitrophenyl chloroformate and monomethoxy poly(ethylene glycol) (mPEG, Mn = 2000) were purchased from Sigma-Aldrich (St. Louis, MO). Doxorubicin hydrochloride (DOX·HCl) was provided by HaiKou Manfangyuan Chemical Company (Haikou, China). Chloroquine (CQ) was acquired from Kaiyang Biotechnology Pharmaceutical (Shanghai, China). Hexachlorocyclotriphosphazene was purchased from Aladdin Ltd. (Shanghai, China) and was sublimated at 90 °C before use. Toluene was dried by refluxing over sodium pieces and distilled just prior to use. Triethylamine (TEA) and petroleum ether were dried over CaH_2_ and distilled just prior to use. All other reagents were commercially available and used as received.

Human chronic myeloid leukemia cancer (K562) cell lines, doxorubicin-resistant human chronic myeloid leukemia cancer (K562/ADR) cell lines, human breast cancer (MCF-7) cell lines and doxorubicin-resistant human breast cancer (MCF-7/ADR) cell lines were provided by Key Gen Biotechnology Co., Ltd. (Nanjing, China). Cells were cultured in RPMI 1640 medium furnished with 10 % fetal bovine serum (Sijiqing Biologic, Hangzhou, China), 100 U/mL penicillin, and 100 μg/mL streptomycin at 37 °C under a humidified atmosphere of 5 % CO_2_. EGFP-LC3 plasmid was provided by Addgene (USA).

Female BALB/c nude mice (15 ± 2 g, 4-5 weeks old) and female Sprague Dawley rats (180 g ± 20 g) were provided by the Laboratory Animal Center of Zhejiang Academy of Medical Sciences (Hangzhou, China), maintained in a pathogenfree laboratory environment and animal experiments were performed in accordance with the Regulations on Experimental Animals of Zhejiang University.

### Synthesis and characterization of poly[(PEG)_x_(ABD)_y_phosphazene]_n_ (PPAP)

The amphiphilic poly[(PEG)_x_(ABD)_y_phosphazene]_n_ copolymer (PPAP) was synthesized by sequentially grafting NH_2_-mPEG_2000_ and 4-aminomethyl-2-benzyloxy-[1,3]-dioxolan (ABD) onto a poly(dichlorophosphazene) backbone through nucleophilic substitution. The chemical structures of PPAP and ABD were confirmed by ^1^H NMR spectroscopic measurements (DMX-500, Brulcer Co., Zurich, Switzerland) using CDCl_3_ as a solvent and FT-IR spectrometer (Nicolet 6700, Thermo Fisher scientific LLC, NY). The more information is clarified in **Supplementary Material (Supplementary section 1)**.

### Drug loading and characterization

The drug-loaded nanovesicles were prepared using membrane dialysis method. Briefly, 8 mg of PPAP was dissolved with 1.6 mg of cholesteryl hemisuccinate in 0.2 mL of DMF, and the equal volume of DOX·HCl aqueous solution was added dropwise under stirring. Then, the mixture was sealed in a dialysis bag (molecular weight cutoff (MWCO): 8k-14kDa) and dialyzed against deionized water for 6 h with frequent change of deionized water. DOX·HCl loaded nanovesicles (D-DIV/C) were obtained after filtered by 0.45 μm filter. DOX·HCl and CQ co-loaded nanovesicles with cholesteryl hemisuccinate (DC-DIV/C) and without cholesteryl hemisuccinate (DC-DIV) were prepared in the same way except that CQ was also dissolved in DOX·HCl solution. The particle size was evaluated by dynamic light scattering (DLS, Malvern Nano-S90, U.K.). The morphologies of various nanovesicles were observed by transmission electron microscopy (TEM, JEM-1200EX, Japan) with an 80 kV accelerating voltage and confocal laser scanning microscope (CLSM, Nikon, Japan). The drug payload was examined by UV-vis spectrophotometry (PuXi TU-1800PC, China) with absorption wavelength monitored at 480 nm for DOX·HCl and 330 nm for CQ 28, 31. Then the loading content (LC, %) and encapsulation efficiency (EE, %) were calculated according to the following equations: Loading content (%) = (Weight of drug in nanovesicles / Weight of drug-loaded nanovesicles) ×100%, Encapsulation efficiency (%) = (Weight of drug in nanovesicles / Weight of feeding drug) ×100%. Moreover, the stability of DC-DIV/C in saline within 48 h was investigated. Meanwhile, the morphology and particle size of DC-DIV/C incubated in PBS at pH 5.5 for 1 h was investigated by TEM and DLS, respectively. In addition, to verify the interaction between drug and PPAP, the infrared spectrum of DOX·HCl (or CQ), ABD, and mixture of ABD and DOX·HCl (or CQ) were measured on a FT-IR spectrometer.

### In vitro drug release

The DOX·HCl and CQ release of nanovesicles were evaluated at different pHs by dialysis method. Specifically, 600 µL of a sample solution was transferred into a dialysis bag (molecular weight cutoff (MWCO): 8k-14kDa), which was immersed in 10 mL of PBS (pH 7.4 and 5.5) at 37 °C with shaking at 100 rpm. At predetermined time intervals, 1mL of buffer solution was withdrawn and replenished with an equal volume of fresh PBS. The amount of DOX·HCl and CQ in the release media was determined using a UV-vis spectrometer. All samples were analyzed in triplicate.

### Cytotoxicity and apoptosis assay

The cytotoxicity of free DOX·HCl, free CQ, physical mixture of free DOX·HCl and CQ (DC), physical mixture of PPAP and cholesteryl hemisuccinate (PPAP/C) and drug-loaded nanovesicles on MCF-7 cells, MCF-7/ADR cells, K562 cells or K562/ADR cells was investigated at 48 h via CCK-8 kit. Accordingly, the reversal index (RI) of DOX·HCl for MCF-7/ADR cells and K562/ADR cells was calculated using the following formula: RI = IC_50_ of the test formulation / IC_50_ of free DOX·HCl. The combination index (CI) between DOX⋅HCl and CQ at different ratios was calculated as follows: CI = D1/(Dm)1 + D2/(Dm)2 + D1*D2/(Dm)1*(Dm)2, where D1 and (Dm)1 represent the IC_50_ values of DOX⋅HCl applied in combination or alone, respectively, and D2 and (Dm)2 represent the IC_50_ values of CQ applied in combination or alone, respectively. In addition, apoptosis analysis was implemented using the Annexin V-FITC/PI kit (LianKe, China) based on the kit instructions. The details were provided by **Supplementary Material (Supplementary section 1)**.

### Cell uptake

The cells were seeded in 12-well plates (2×10^5^ cells/well) and incubated for 24 h in 1ml RPMI 1640 containing 10 % fetal bovine serum. Then the cells were incubated in the medium containing various formulations (with an equivalent DOX·HCl concentration of 10 µg/mL). After the appropriate period, the cells were washed with precooling PBS (pH 7.4) three-times, collected, and resuspended in PBS. Finally, the suspension was analyzed by a flow cytometry (Cytoflex, Beckman, U.S.) with the 488 nm argon ion laser. The individual fluorescence of 10^4^ cells was collected for each sample at the appropriate period. Also, the intracellular amount of DOX·HCl and CQ after the incubation of free DC and DC-DIV/C were determined by a multifunctional microplate reader (SpectraMax M5, USA) as the two drugs in various cells were extracted by the mixture of methanol and water (v/v=1/1). All experiments were run in triplicate. P-gp expression and its functional activity was evaluated as described in **Supplementary Material (Supplementary section 1)**.

### Autophagy assays

MCF-7/ADR and K562/ADR cells were transfected with EGFP-LC3 plasmid (Addgene, USA) by means of Lipofectamine 2000 (Invitrogen, USA) according to the manufacturer's protocol. The transfected cells were incubated with CQ (10 µg/mL) for 24 h. Afterwards, cells were washed with precooled PBS, then the cell nuclei and lysosomes were labeled with Hoechst 33342 (Invitrogen, USA) and LysoTracker^®^ Red DND-99 (Invitrogen, USA), respectively, for confocal laser scanning microscope (CLSM, Olympus, Japan) observation. In addition, some transfected cells were incubated with various formulations for 24 h at the final DOX·HCl and CQ concentration of 5 µg/mL and 10 µg/mL, respectively. Then, these cells were processed with the same operation as described above and cell nuclei were labeled with Hoechst 33342. The untreated or treated cells were observed with CLSM after being fixed. Furthermore, confocal microscopy analysis was used to measure the fluorescent dots representing EGFP-LC3 translocation per cell.

Moreover, the expression of autophagy related marker protein LC3 and autophagy substrate protein p62 was detected using western blot. The cellular autophagy was also investigated by bio-TEM. The details were described in **Supplementary Material (Supplementary section 1)**.

### Pharmacokinetic study

Female Sprague Dawley rats (180 g ± 20 g) were divided into three groups (*n* = 3) and intravenously administered free DC, D-DIV/C, and DC-DIV/C at the dose of 5 mg/kg of DOX·HCl and 10 mg/kg of CQ. Approximately 0.5 mL of blood samples were collected into heparinized tubes from the orbital venous plexus at 0.083, 0.25, 0.5, 1, 2, 4, 6, 8, 10, 24, 48 and 72 h and then centrifuged at 8000 rpm for 5 min. The plasma samples were harvested and stored at -20 °C until analysis. The plasma concentrations of DOX·HCl and CQ were assayed using high-performance liquid chromatography (HPLC, Shimadu, Japan). The detailed measurement was described in **Supplementary Material (Supplementary section 1)**. The pharmacokinetic parameters of DOX·HCl and CQ in various formulations were calculated from a two-order compartment model using Thermo Kinetica 4.4.1 software.

### In vivo biodistribution

For in vivo biodistribution study, the mice bearing 500 mm^3^ of K562/ADR tumors were intravenously administrated with various formulations respectively. The dose of DOX·HCl maintained at 5 mg/kg. To avoid the signal interference from the animal body, the major organs (heart, liver, spleen, lung, and kidney) and tumors of the sacrificed mice were harvested for fluorescent imaging after 24 h intravenous injection. The fluorescence intensity of the tumors and organs was measured and analyzed using in vivo imaging system (CLS136341/F, PerkinElmer, Germany) with the excitation and emission wavelength at 480 nm and 595 nm, respectively.

### In vivo anti-tumor activity and histological analysis

The BALB/c nude mice were subcutaneously inoculated with K562/ADR cells (2×10^7^ cells) on the right flank to construct K562/ADR tumor-bearing mice models. After 10 days, mice were randomly divided into seven groups (n = 5) once the tumors reached about 80 mm^3^. And then various formulations were injected intravenously through the tail vein every other day for four times and saline was taken as the control group. During 16 days, the body weight and tumor volumes (V(mm^3^) = [length × (width)^2^]/2.) were recorded every 2 days. Later, the mice were sacrificed and the tumors were excised and weighted. In addition, tumors and major organs (heart, liver, spleen, lung, and kidney) were collected for slice analysis by hematoxylin and eosin (H&E) staining. The tumors were also analyzed by immunohistochemical detection of Ki67, immunofluorescence assay of LC3 and terminal deoxynucleotidyl transferased dUTP nick end labeling (TUNEL) assay.

### Statistical analysis

The data were expressed as the mean ± SD. The statistical significance was determined using one-way ANOVA analysis using SPSS 17.0 software.

## Results and Discussions

### Synthesis and self-assemble character of PPAP

In view of our previous work, a transition from random water-soluble chains to self-assembled micelles occurred as *f*_PEG_ was reduced below 0.94 for the amphiphilic polyphosphazenes.^29^ More accurately, the upper *f*_PEG_ is also limited to guarantee the powerful drug loading when accounting for the interaction between DOX·HCl and hydrophobic ABD groups in PPAP. Hence, the amphiphilic PPAP with an appropriate feed ratio was synthesized in this study by sequential substitution reactions of mPEG_2000_-NH_2_ and ABD with chlorine atoms on the poly(dichlorophosphazene) backbone (**Scheme S1**). The structure of ABD was confirmed by ^1^H NMR (500 MHz, CDCl_3_, δ) (**Figure S1**) with the characteristic signals at 2.10 ppm (m, 2H, NH_2_), 2.70-2.86 ppm (m, 2H, N-CH_2_), 3.64-3.76 ppm (m, 1H, OCHCH_2_), 4.01-4.28 ppm (m, 2H, OCHCH_2_), 4.50-4.64 ppm (m, 2H, OCH_2_C_6_H_5_), 5.87-5.93 ppm (m, 1H, OCHO), 7.35-7.4 ppm (m, 5H, C_6_H_5_). The final amphiphilic PPAP was characterized by ^1^H NMR and FT-IR. As **Figure S2B** shows, the characteristic bands of PPAP were observed from the FT-IR spectrum: mPEG_2000_ at 2886 cm^-1^ (-CH_2_- stretching vibration), 1450 cm^-1^ (-CH_2_- deformation vibration), 1105 cm^-1^ (C-O-C stretching vibration), and 1656 cm^-1^ (C=O stretching vibration); ABD at 1533 cm^-1^ (phenyl stretching vibration). Additionally, polyphosphazene backbones at 1351 cm^-1^ (P=N stretching bands) and 954 cm^-1^ (P-N stretching vibration) were also observed. To further identify the chemical structure of PPAP, the characteristic bands of PPAP were observed from the ^1^H NMR (500 MHz, CDCl_3_, δ) spectrum (**Figure S2A**). The characteristic signals of PPAP were displayed as 3.3 ppm (mPEG, 3H, -OCH_3_), 3.6 ppm (mPEG, 4H, -OCH_2_CH_2_-), and 7.35-7.4 ppm (ABD, 5H, phenyl). By comparing the peak area at 3.3 ppm of mPEG and that at 7.35-7.4 ppm of ABD, the mole ratio of mPEG_2000_/ABD (x/y) was obtained, namely, x/y=1.60/0.40. The weight ratio of mPEG (f_PEG(w)_) in PPAP could be calculated using the following equation: *f*_PEG(w)_ = M_PEG_ × x/(M_PEG_ × x + M_ABD_ × y +M_PPP_), where M_PEG_, M_ABD_, and M_PPP_ represent the molar masses of mPEG_2000_, ABD, and repeated units (N=P) of the polyphosphazene backbone, respectively. The result of *f*_PEG(w)_ was calculated as 0.96. These characterizations indicated that PPAP was synthesized with the desired structure.

**Figure 1:**
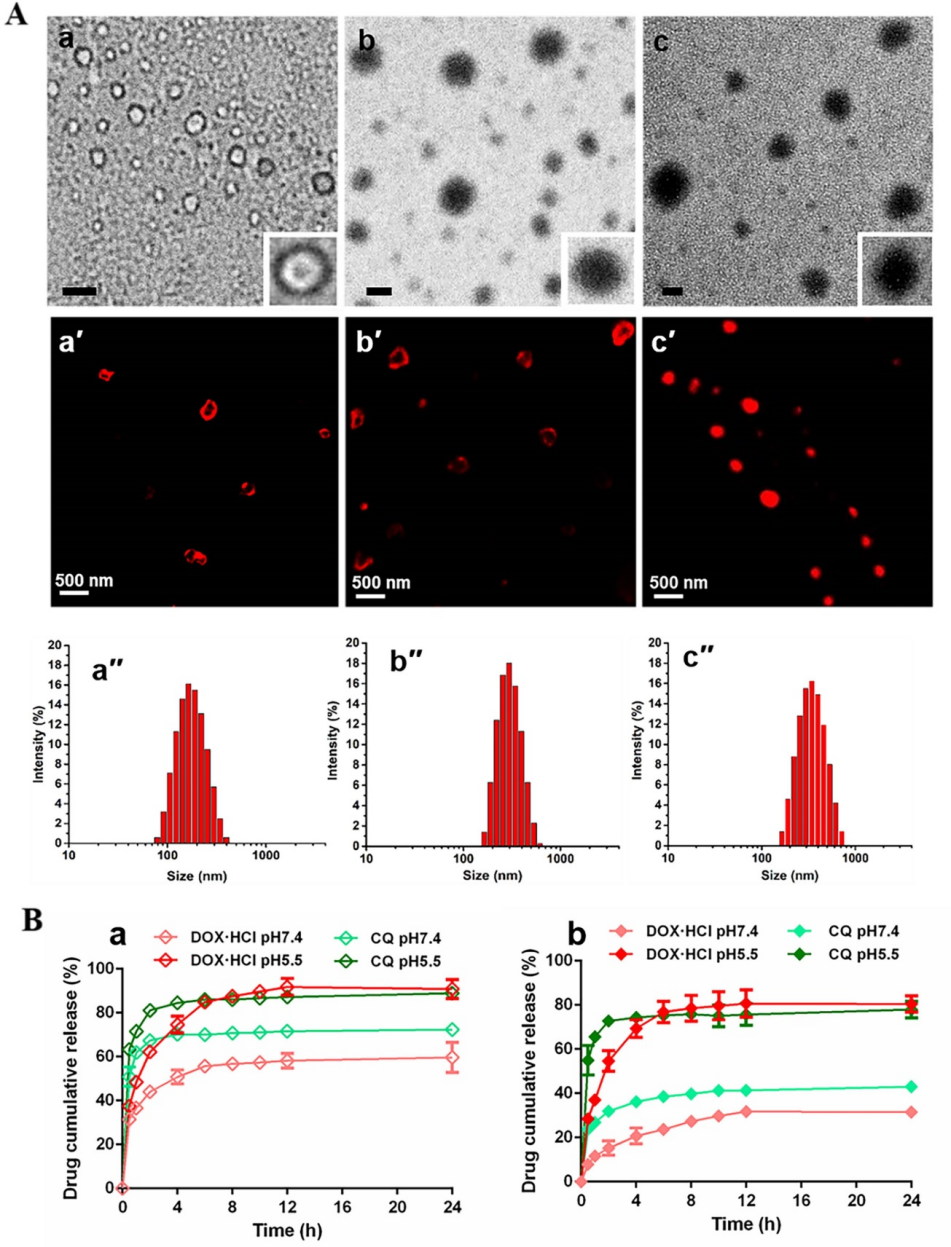
Characterization of nano-formulations. (A) TEM images of (a) DIV (∼7 % theoretical LC of DOX·HCl), (b) DC-DIV/C and (c) D-DIV/C (∼20 % theoretical LC of DOX·HCl), all scale bars are 0.2 µm; CLSM images of (a′) DIV (∼7 % theoretical LC of DOX·HCl), (b′) DC-DIV/C and (c′) D-DIV/C (∼20 % theoretical LC of DOX·HCl). (B) In vitro drug release profiles of (a) DC-DIV and (b) DC-DIV/C at pH 7.4 and 5.5.

TEM and CLSM was used to evaluate the self-assemble character and drug loading of PPAP. PPAP itself cannot form nanoparticles in any shape. The similar phenomenon occurred when CQ was mixed with PPAP during dialysis (**Figure S3Cb**). However, nanoparticles possessing typical vesicular morphology with an aqueous cavity could be observed in **Figure 1Aa and a′** as a little amount of DOX·HCl (∼7 % theoretical LC) replaced CQ, indicating drug-induced self-assembled nanovesicle (DIV) was successfully accomplished. Further increasing the feed amount of DOX·HCl (∼20 % theoretical LC), the vesicle became solid nanoparticles since some DOX·HCl filled in the center cavity even when cholesteryl hemisuccinate was meanwhile added into the organic solution during dialysis to get D-DIV/C (**Figure 1Ac, c′**). As compared with FT-IR spectrum of ABD and CQ (**Figure S3B**), the characteristic peak (3366 cm^-1^) of the amino group in ABD of PPAP became flat and moved to 3332 cm^-1^ as ABD was mixed with DOX·HCl **(Figure S3A)**, showing that there existed hydrogen bond interaction between hydrophobic group ABD in PAPP and DOX·HCl. This difference verified the reason why DOX·HCl was able to render DIV formation but CQ couldn't. Then, the dual drug loading capacity was investigated on the premise of DIV formation. To be noticed, we lowered the feed amount of DOX·HCl (∼7 % theoretical LC) to meet the requirement of DIV formation as well as to release more space for CQ loading. The resultant DOX·HCl and CQ co-loaded nanovesicle named as DC-DIV/C, displayed solid particles morphology (**Figure 1Ab**). Also, CQ was basically encapsulated in the hydrophilic cavity of nano-vesicles owing to the vesicular membrane driven by DOX·HCl which displays a brilliant fluorescent circle in CLSM picture (**Figure 1Ab′**) like that of DIV (∼7 % theoretical LC of DOX·HCl) (**Figure 1Aa′**). The actual LC of DOX·HCl and CQ in DC-DIV/C were 4.6 % (EE 77.4 %) and 8.7 % (EE 43.3 %), respectively (**Table S1**). If cholesteryl hemisuccinate was absent in the preparation process, DOX·HCl and CQ could be also co-loaded to obtain DC-DIV with 4.3 % and 8.7 %. In addition, the stability of DC-DIV/C in saline was investigated. Both DLS data and TEM images demonstrated that DC-DIV/C exhibited stable particle size and PDI within 48 h (**Figure S4**). Meanwhile, the actual LC of DOX·HCl and CQ in DC-DIV/C within 48 h were maintained almost the same as the initial value (**Table S2**).

### Drug release behaviors of DC-DIV and DC-DIV/C

The in vitro drug release behaviors of DC-DIV and DC-DIV/C were studied under pH 7.4 and pH 5.5 conditions, respectively. As shown in **Figure 1Ba**, the accumulative release ratios of DOX·HCl and CQ from DC-DIV exhibited a sharp premature drug leakage, reaching 32 % and 51 % within 0.5 h at pH 7.4, respectively. And finally, over 60 % of DOX·HCl and 72 % of CQ were released after 24 h, implying the potential toxicity and inadequate DOX·HCl and CQ arriving tumor site during the in vivo circulation. Fortunately, once cholesteryl hemisuccinate, joined into the formulation as DC-DIV/C, DOX·HCl and CQ released about 30 % and 40 % at 24 h (**Figure 1Bb**), revealing that cholesteryl hemisuccinate could simultaneously inhibited the premature leakage of both water-soluble cargos under normal physiological conditions. This obvious plugging effect will effectively prevent side effects caused by premature leakage and maintain the drug co-loading ratio during circulation 32, 33. Furthermore, when exposed to pH 5.5, a sharply accelerated and entire drug release was displayed. It has been validated that the ortho ester ABD in PPAP could be hydrolyzed into small molecule formic acid and alcohol under acidic conditions via the cleavage of five-membered cyclic ortho ester following an exocyclic mechanism 29, 34-36. The TEM image and DLS result of DC-DIV/C incubated in PBS at pH 5.5 for 1 h showed the particle size and PDI increased significantly to 695.4 nm and 0.946 (**Figure S3Cc and S3Cd**). Therefore, the pH-sensitive drug release character could be attributed to the hydrolysis of the ortho ester ABD in PPAP, as well as the subsequent swelling and rupture of DC-DIV and DC-DIV/C. In brief, as a carrier for dual water-soluble cargos, DC-DIV/C could restrain premature leakage of the two water-soluble drugs in the meantime during blood circulation and supposed to perform an acid-sensitive synchronous drug release behavior in tumor cells, displaying its superiority to DC-DIV and paving the way for the synchronous delivery of DOX⋅HCl and CQ to the target site at the optimal ratio.

### Cytotoxicity and cell apoptosis

**Figure 2:**
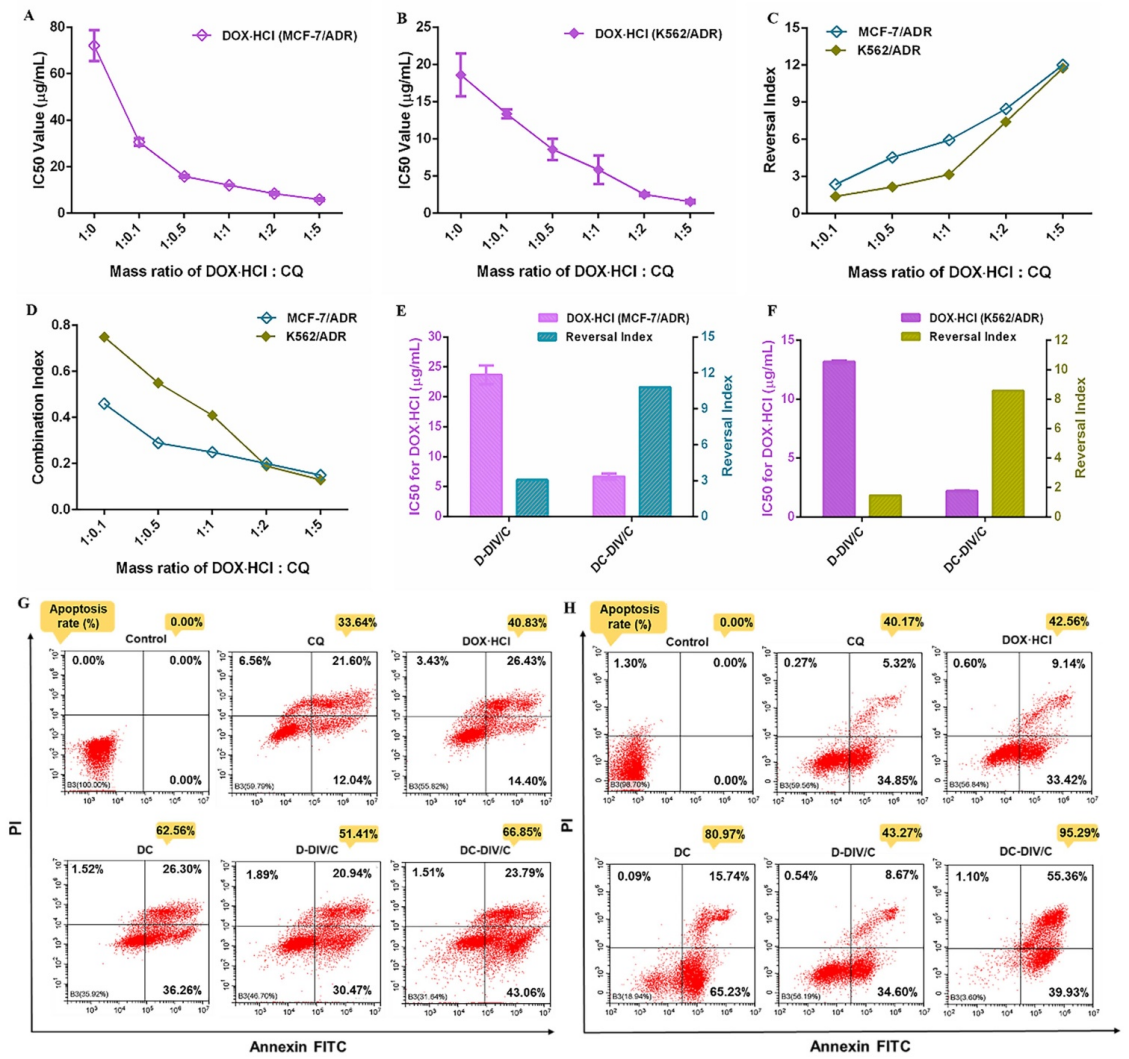
IC_50_ values of DOX⋅HCl against (A) MCF-7/ADR cells and (B) K562/ADR cells in the form of free drug mixtures with CQ at different mass ratios; (C) Reversal index of DOX⋅HCl and (D) combination index of DOX⋅HCl and CQ at different mass ratios on MCF-7/ADR and K562/ADR cells (CI<0.1, very strong synergism; CI = 0.1-0.3, strong synergism; CI = 0.3-0.7, synergism; CI = 0.7-0.85, moderate synergism; CI = 0.85-0.90, slight synergism; CI = 0.90-1.10, nearly additive; CI =1, additive; CI>1, antagonistic.); IC50 values of DOX⋅HCl and reversal index after treated with D-DIV/C or DC-DIV/C against (E) MCF-7/ADR cells and (F) K562/ADR cells; Apoptosis of (G) MCF-7/ADR cells and (H) K562/ADR cells after treatment with different formulations for 48 h.

The in vitro cell viability was investigated and showed that the IC_50_ of free DOX·HCl on MCF-7/ADR and K562/ADR cells were 72.12 μg/mL and 18.91 μg/mL (**Figure S5C and S5F**), respectively, which supported the DOX·HCl-resistance index of MCF-7/ADR and K562/ADR cells was 902-fold and 126-fold. Interestingly, the addition of CQ significantly enhanced the sensitivity of drug-resistant cells to DOX·HCl. The IC_50_ of DOX⋅HCl on MCF-7/ADR and K562/ADR cells were obtained after being treated with the physical mixture of free DOX⋅HCl and CQ in various ratios (**Figure 2A, B**), and then the reversal index (RI) and combination index (CI) between the two drugs was calculated, respectively (**Figure 2C, D**). For MCF-7/ADR and K562/ADR cells, the IC_50_ of DOX·HCl decreased significantly as increasing the proportion of CQ. Correspondingly, the RI values showed a rapid growth and finally increased to about 12 along with the increasing proportion of CQ, and meanwhile the CI values exhibited a significant decline. As a dominant parameter to characterize synergism, CI value deceased to a range of 0.1 - 0.3 as the mass ratio of DOX·HCl and CQ was 1:2 or 1:5, indicating a strong synergistic effect according to the scoring criteria 37. Therefore, the mass ratio of 1:2 was chosen as the reasonable ratio for the combination formulation of DOX·HCl and CQ. Also, as discussed above, this optimal co-loading ratio could be guaranteed by DC-DIV/C (**Table S1**).

Ulteriorly, the cytotoxicity of various nanoparticles was investigated (**Figure S5**). With the application of D-DIV/C on MCF-7/ADR and K562/ADR cells, the IC_50_ of DOX·HCl decreased from 72.12 μg/mL and 18.91 μg/mL to about 24 μg/mL and 13 μg/mL, which made the RI values get to 3.0 and 1.4, respectively (**Figure 2E, F**). Satisfactorily, DC-DIV/C showed much more excellent lethal effect. For DC-DIV/C, the IC_50_ of DOX·HCl on MCF-7/ADR and K562/ADR cells severely decreased to 6.68 μg/mL and 2.21 μg/mL, respectively. And meanwhile the RI values soared to 10.80 and 8.56. Furthermore, the IC_50_ values of DOX⋅HCl generated by the given DC-DIV/C were evidently lower than that of DC. Likewise, the RI values produced by DC-DIV/C were significantly higher than that of DC. These results indicated that DIV/C-based DOX⋅HCl and CQ co-delivery system displayed more wonderful cytotoxicity than DC. Besides, the cell viability of PPAP/C on MCF-7/ADR and K562/ADR cells were over 90 % (**Figure S6**), which revealed that neither of them would affect the in vitro efficacy evaluation of synergistic drug delivery. Cytotoxicity data of non-drug resistant MCF-7 and K562 cells are shown in **Figure S5G, H** and **Table S3**. As expected, the cytotoxicity of both D-DIV/C and DC-DIV/C failed to display any strengthening effect on MCF-7 and K562 cells when compared with free DOX⋅HCl since the two cells are inherently sensitive to DOX⋅HCl. Equally, PPAP/C had no interference on the cytotoxicity of D-DIV/C and DC-DIV/C (**Figure S6**).

Also, DC-DIV/C stimulated pro-apoptotic effect on MCF-7/ADR and K562/ADR cells. As shown in **Figure 2G and H**, while keeping the same dosage of 5 μg/mL DOX⋅HCl of various formulations for 48 h, DC-DIV/C triggered more than 66 % apoptosis on MCF-7/ADR cells, exhibiting noticeable contrast with 33.64 % of free CQ, 40.83 % of free DOX⋅HCl, 51.41 % of D-DIV/C and 62.56 % of free DC. The same trend also appeared for the K562/ADR cells on the premise of the same dosage and treatment time. The apoptosis rate of different formulations containing free CQ, free DOX⋅HCl, free DC, D-DIV/C and DC-DIV/C were 40.17 %, 42.56 %, 80.97 %, 43.27 % and 95.29 %, separately. These outcomes not only demonstrated CQ itself induced slight apoptosis at a high safe dose, but also confirmed the central role of CQ in the process of synergistic anti-tumor action.

### Cellular uptake and synchronism investigation

**Figure 3:**
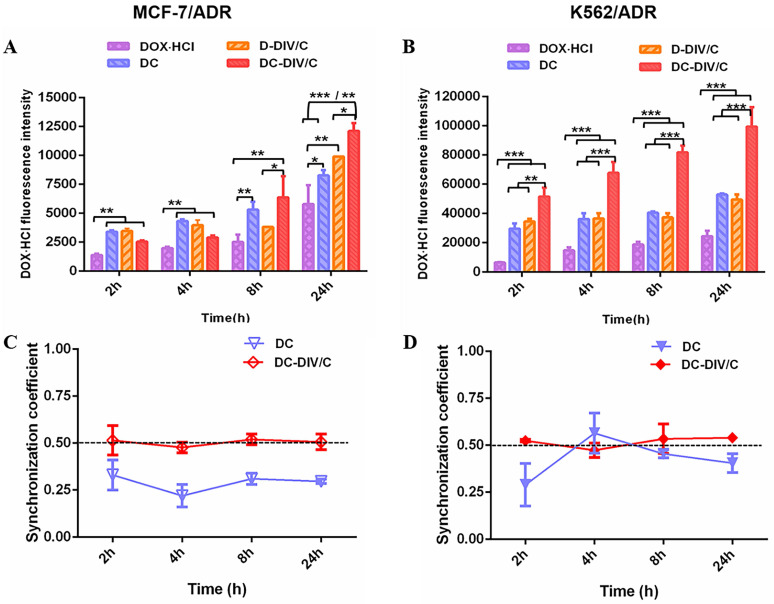
Cellular uptake of DOX·HCl and uptake synchronism in doxorubicin-resistant cells. (A-B) DOX·HCl cellular accumulation in MCF-7/ADR (A) and K562/ADR (B) cells at different points were quantitatively determined by flow cytometry. The concentration of DOX·HCl and CQ in these various groups were 5 µg/ml and 10 µg/mL, respectively. (C-D) Synchronization coefficient (the ratio of DOX·HCl / CQ) exhibited by (C) MCF-7/ADR cells and (D) K562/ADR cells. (*p<0.05, **p<0.01, ***p<0.001, mean ± SD, n =3).

The enhanced cytotoxicity and cell apoptosis induced by DC-DIV/C might be partially attributed to the improved DOX⋅HCl internalization. Thus, cellular uptakes of different formulations were determined using flow cytometry (Cytoflex, Beckman, U.S.) by virtue of inherent fluorescence of DOX⋅HCl. The fluorescence intensity of DOX·HCl exhibited the growth trend over time on sensitive cells (MCF-7 and K562) without individual difference after the treatment of various formulations including free DOX·HCl, D-DIV/C, DC-DIV/C, and free DC** (Figure S7)**. However, as shown in **Figure 3A and B**, the final fluorescence intensity of DOX⋅HCl derived from DC was about 1.33 and 2 times higher than that of free DOX·HCl in MCF-7/ADR (*p<0.05) and K562/ADR (***p<0.001), respectively. Evidently, this stronger fluorescence was also observed for DC-DIV/C when compared with D-DIV/C in MCF-7/ADR (*p<0.05) and K562/ADR (***p<0.001). And the fluorescence of drug-loaded nanovesicle (D-DIV/C or DC-DIV/C) was higher than that of the corresponding free drug (DOX⋅HCl or DC). These differences indicated that polymeric nanocarrier and CQ were crucial factors in the process. Our previous studies confirmed that polyphosphazene-based nanoparticles were predominantly captured by means of endocytosis process 38, 39. Then CQ is able to weaken P-glucose protein (P-gp) activity to inhibit the efflux of DOX·HCl as a substrate 37, thus increasing the intracellular drug concentration to acquire drug resistance reversal. **Figure S8A and B** show, the extremely high expression of P-gp existed in MCF-7/ADR cells and K562/ADR cells, but none in MCF-7 cells and K562 cells. Meanwhile, the P-gp expression in drug-resistant cells almost didn't change after treated with various formulations. However, as a classical P-gp substrate, more and more rhodamine 123 was accumulated in drug-resistant cells when increasing CQ concentrations (**Figure S8C and D**), confirming that CQ can inhibit the efflux activity of P-gp 37.

To further explore the synchronism on cellular uptake of DOX·HCl and CQ, the quantification analysis of the intracellular concentration of two drugs in MCF-7/ADR and K562/ADR cells were recorded. As shown in **Figure 3C and D**, the intracellular ratios of DOX·HCl and CQ with the treatment of free DC were obviously deviated from the optimal ratio (namely, DOX·HCl / CQ = 0.5), exhibiting irregular tendency as time went by. In contrast, DC-DIV/C maintained a synchronous rhythm in consistent with the optimum ratio of 0.5 over time. This phenomenon proved the necessity of nano-carriers in the synchronous delivery of DOX·HCl and CQ, also foreshowing potentiality of DC-DIV/C for in vivo anti-tumor therapy.

### Autophagic level analysis

**Figure 4:**
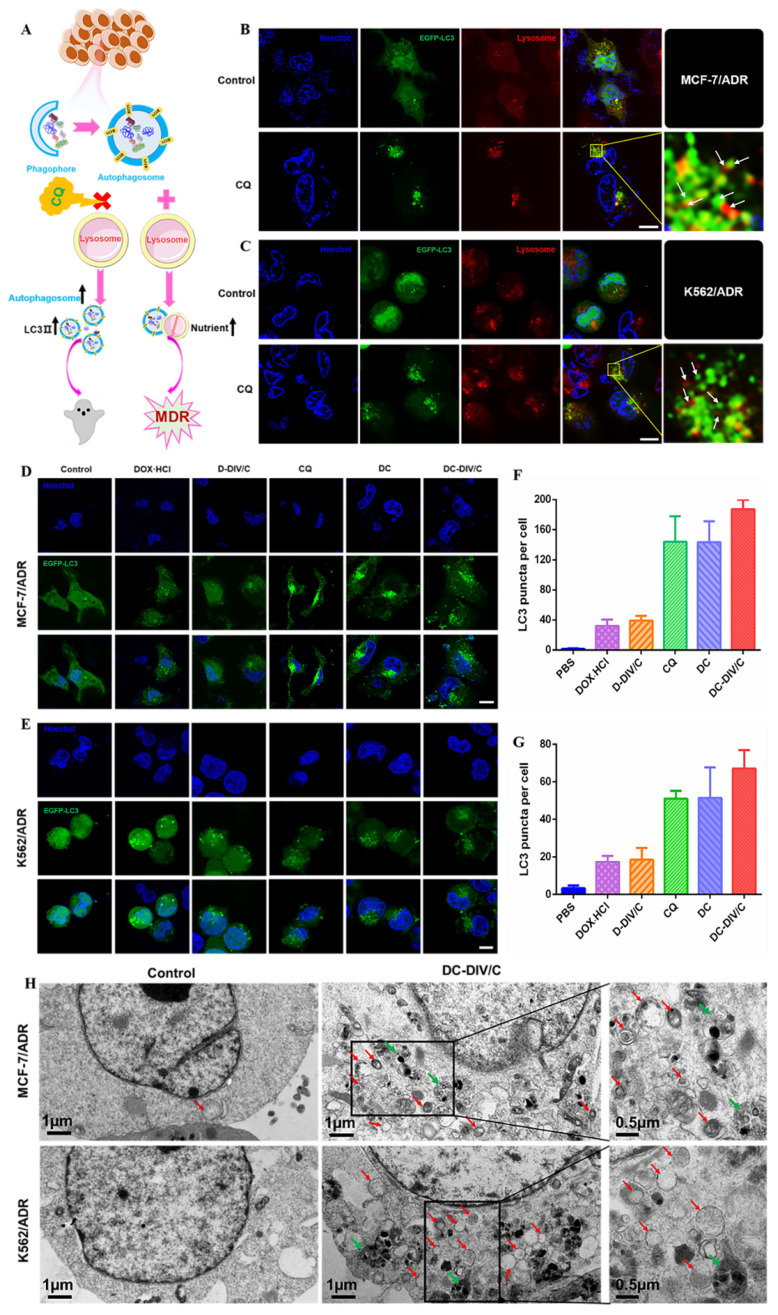
Intracellular autophagic level and autophagic flux analysis. (A) A simple schematic diagram on the role of autophagy inhibition with CQ against drug resistant cells. (B-C) EGFP-LC3 transfected MCF-7/ADR (B) and K562/ADR (C) cells were treated with 10 µg/ml CQ for 24 h, the lysosome detected with Lyso-Tracker Red probes. (D-E) Representative images of EGFP-LC3 transfected MCF-7/ADR (D) and K562/ADR cells (E). The EGFP-LC3 transfected cells were treated with various groups for 24 h and the concentration of DOX·HCl and CQ in these various groups were 5 µg/ml and 10 µg/ml, respectively. (F-G) Quantification of EGFP-LC3 puncta in MCF-7/ADR (F) and K562/ADR (G) cells. All scale bars: 10 µm. (H) TEM images of MCF-7/ADR cells and K562/ADR cells treated with PBS and DC-DIV/C for 48 h (red arrows indicate autophagosomes; green arrows indicate autolysosomes). Data are shown as the mean ± SD.

**Figure 5:**
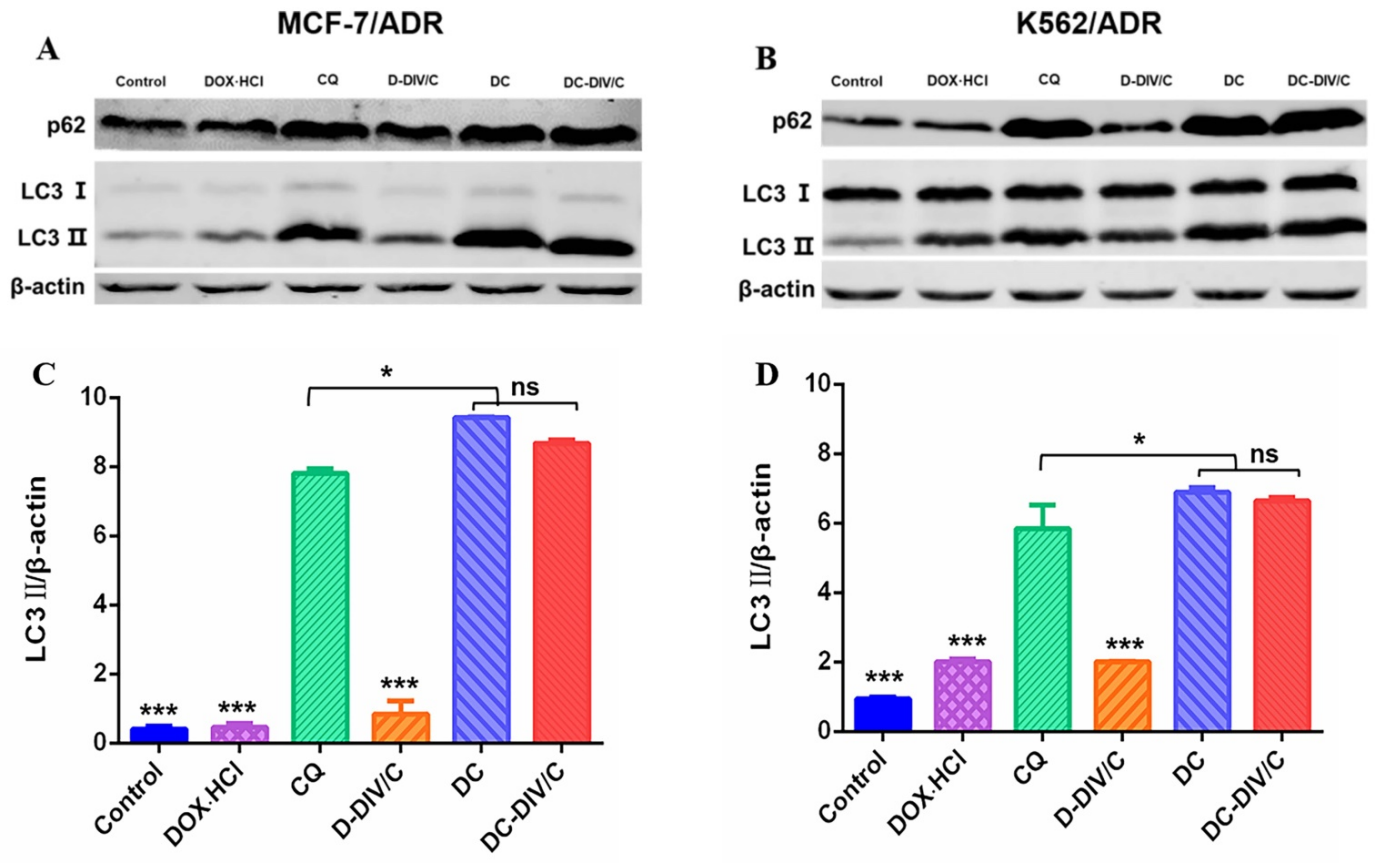
Changes of autophagy marker protein in doxorubicin-resistant cells. Western blot analysis of p62 and LC3 protein expression in (A) MCF-7/ADR cells and (B) K562/ADR cells. Densitometric analysis of LC3-II level in (C) MCF-7/ADR cells and (D) K562/ADR cells. (*p<0.05, ***p<0.001, mean ± SD, n = 3).

As reported, activated autophagy could be another factor to induce drug resistance 40, 41, so that the autophagic level of various formulations was investigated considering CQ is a classic autophagy inhibitor. As a weak base, CQ can be diprotonated and entrapped in lysosomes, then inhibits the lysosomal activity via increasing the pH in lysosomes 42, restraining the degradation of the autophagic cargos, blocking the fusion of autophagosomes with lysosomes, thus numerous autophagosomes were accumulated in the cytoplasm (**Figure 4A**). To monitor the blockade of autophagic flux and accumulation of autophagosomes, the expressed fluorescent autophagy marker EGFP-LC3 was utilized as reported 43. The assessment of autophagy is made by measuring membrane-conjugated EGFP-LC3 presented on autophagosomes (the green fluorescent spots, EGFP-LC3 puncta). Firstly, as shown in **Figure 4B and C**, autophagosomes (the green fluorescent spots) and lysosomes (the red fluorescent spots) were basically separated in MCF-7/ADR and K562/ADR cells after the application of CQ, confirming the blocking effect of CQ on the fusion between autophagosomes and lysosomes. To verify the same effect of DC-DIV/C, the cellular TEM was implemented (**Figure 4H**), it can be observed from the TEM images that numerous autophagosomes (red arrows) and a few autolysosomes (green arrows) accumulated in the DC-DIV/C group, in contrast to the control group (PBS treatment), indicating that the fusion of autophagosomes and lysosomes was blocked by DC-DIV/C. Based on the blockade of fusion between autophagosomes and lysosomes, **Figure 4D and E** show that substantial autophagosomes (the green fluorescent spots, EGFP-LC3 puncta) were accumulated in the cytoplasm as the drug resistant cells treated with the formulations containing CQ (CQ, DC and DC-DIV/C) for 24 h. As the numbers of puncta per cell were quantified by counting software and averaged over three separate experiments, we observed that the formulations containing CQ caused an approximately three-fold increase in the number of EGFP-LC3 puncta (**Figure 4F, G**). The expression of autophagy related marker protein LC3 and autophagy substrate protein p62 was detected using western blot 44, 45. As **Figure 5A and B** show, the protein LC3 displayed a trait that a massive accumulation of autophagosomal membrane-bound LC3-II occurred in MCF-7/ADR and K562/ADR cells after the treatment of the formulations containing CQ for 48 h due to the interrupted autophagy process. As a marker protein of autophagy, autophagosomal membrane-bound LC3-II can be transformed from cytoplasmic LC3-I, and the high level of LC3-II reflected the increase in the amount of autophagosomes. Accordingly, the ratios of LC3-II/β-actin presented by the formulations containing CQ also exhibited a notable increase compared with the formulations containing DOX·HCl alone or control group (***p<0.001). Meanwhile, DC and DC-DIV/C exhibited the higher ratios of LC3-II/β-actin compared with those presented by free CQ (*p<0.05) whether in MCF-7/ADR or K562/ADR cells (**Figure 5C, D**). Since the autophagy substrate protein p62 can be wrapped into autophagosomes and then degraded by protease in autolysosomes, the elevated p62 level is usually considered as a sign of the inhibition of autophagy activity. The high expression of p62 was shown in the formulations containing CQ, further indicating the degradation of autophagic cargo and autophagy activity was significantly inhibited by CQ, DC, as well as DC-DIV/C. Taken together, TEM and CLSM images revealed the blocking effect of CQ on the fusion between autophagosomes and lysosomes by elevating lysosomal pH based on the intrinsic lysosomal tropism of CQ. Meanwhile, the high expression of LC3Ⅱ and p62 in drug resistant tumor cells treated with formulations containing CQ indicated the downstream of autophagy was blocked and the fusion of autophagosomes and lysosomes could not proceed. As a result, the autophagy process could not be completed, and then the drug resistant tumor cells would fail to cope with the metabolic stress and finally undergo apoptosis with the help of chemotherapy. Therefore, autophagy inhibition is expected to improve the efficiency of chemotherapy in MDR cancer via hindering the lysosomal function with the weak base drug CQ.

### In vivo pharmacokinetic behavior and biodistribution

**Figure 6:**
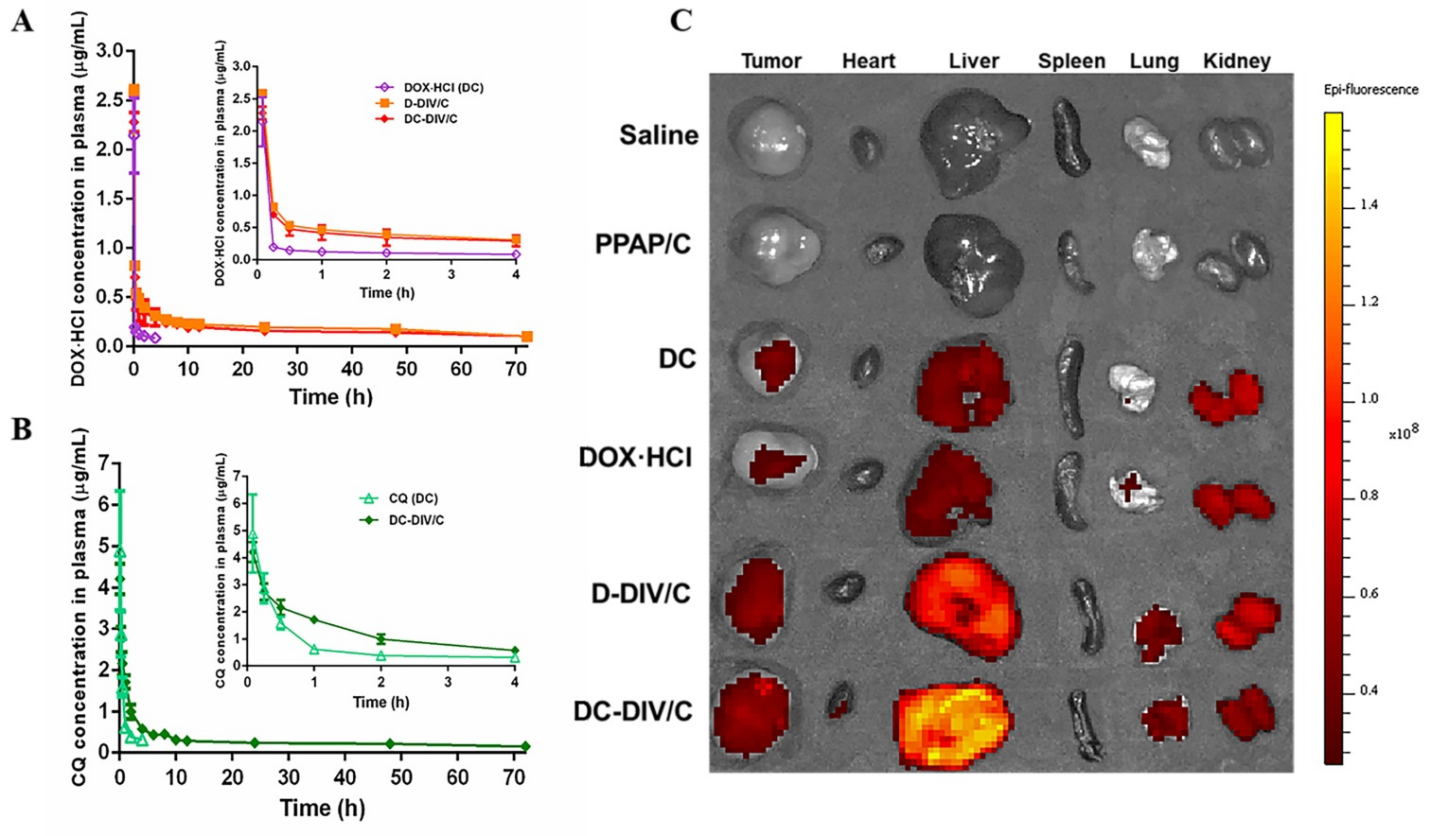
(A) Plasma concentration-time profiles of DOX·HCl after intravenous injection of free DC, D-DIV/C and DC-DIV/C at 5 mg/kg DOX·HCl in rats; (B) Plasma concentration-time profiles of CQ after intravenous injection of DC and DC-DIV/C at 10 mg/kg CQ in rats. (C) The ex vivo fluorescence images of the dissected organs and tumors of different groups at 24 h post-injection.

Then we performed the in vivo pharmacokinetics of various formulations to investigate the fate of DOX·HCl and CQ in the blood. The plasma concentration-time curves are shown in **Figure 6A and B**. Corresponding pharmacokinetic parameters are summarized in **Table 1**. The plasma concentration of free DOX·HCl and free CQ decreased obviously after i.v. injection, and the noticeable difference between them was that the plasma concentration of free DOX·HCl decreased much faster than that of free CQ, which led to the failure of the two drugs to maintain the optimal administration ratio in the blood circulation, thereby affecting the tumor inhibition effect. To get out of this dilemma, the application of vehicles was necessary. Just as shown, evidently, the drug concentration derived from D-DIV/C and DC-DIV/C in plasma was significantly higher than that of the free DC at almost all time periods and exhibited an extensively prolonged circulation time. Furthermore, the plasma concentration of CQ derived from DC-DIV/C was almost twice as much as that of DOX·HCl, which was basically consistent with the optimal ratio of the two drugs. And the pharmacokinetic parameters further verified these above results. As shown in **Table 1**, half-lives (*t*_1/2β_) of D-DIV/C and DC-DIV/C were extended to 37.72, and 33.70 h, respectively, which were about 8.35, and 7.46-fold longer than free DOX·HCl. The area under the time-concentration curve (AUC) data also revealed a similar trend. Obviously, the AUC of D-DIV/C and DC-DIV/C was 13.56 μg·h/mL and 12.62 μg·h/mL, which was about 13.17-fold and 12.25-fold higher than free DOX·HCl, respectively. D-DIV/C and DC-DIV/C all exhibited the longer MRT in vivo along with shorter total body clearance (CL) compared with free DOX·HCl. Moreover, *t*_1/2β_ of DC-DIV/C was 4.79-fold longer, the AUC was 6.47-fold higher, the MRT was 5.61-fold longer and the CL was observably shorter than that of free CQ** (Table 1)**. Notably, for DC-DIV/C, the AUC of CQ was almost twice times as much as that of DOX·HCl, correcting the dislocation incurred by free DC with the same administration ratio. Meanwhile, DC-DIV/C was basically guaranteed the consistency of DOX·HCl and CQ in *t*_1/2β_ or MRT. Based on the above-mentioned evidence, DC-DIV/C with ensured ability to deliver the two drugs synchronously in vivo, providing a foundation for the efficient anti-tumor effect.

Furtherly, the ex vivo fluorescence images of various groups were taken (**Figure 6C**) to investigate the distribution, especially the tumor accumulation of DOX·HCl. The results clarified that the intratumoral DOX·HCl accumulation of drug-loaded DIV/C was significantly more than that of free DOX⋅HCl and free DC due to EPR effect. Whereas free DOX·HCl and free DC exihibited less tumor distribution of DOX⋅HCl owing to the rapid metabolism verified by in vivo pharmacokinetics. Furtherly, free DC showed a little more tumor distribution of DOX⋅HCl compared to free DOX⋅HCl in view of the existence of CQ. Herein, DC-DIV/C exhibited the most abundant DOX⋅HCl accumulation. To be noticed, it seems difficult to avoid drug distribution in the liver, which maybe a common problem for nanoparticles with passive targeting feature. Though the point of targeting efficiency isn't a key issue of this study, the modification on DC-DIV/C with certain active tumor targeting groups would be considered.

### In vivo antitumor activity and toxicity evaluation

**Figure 7:**
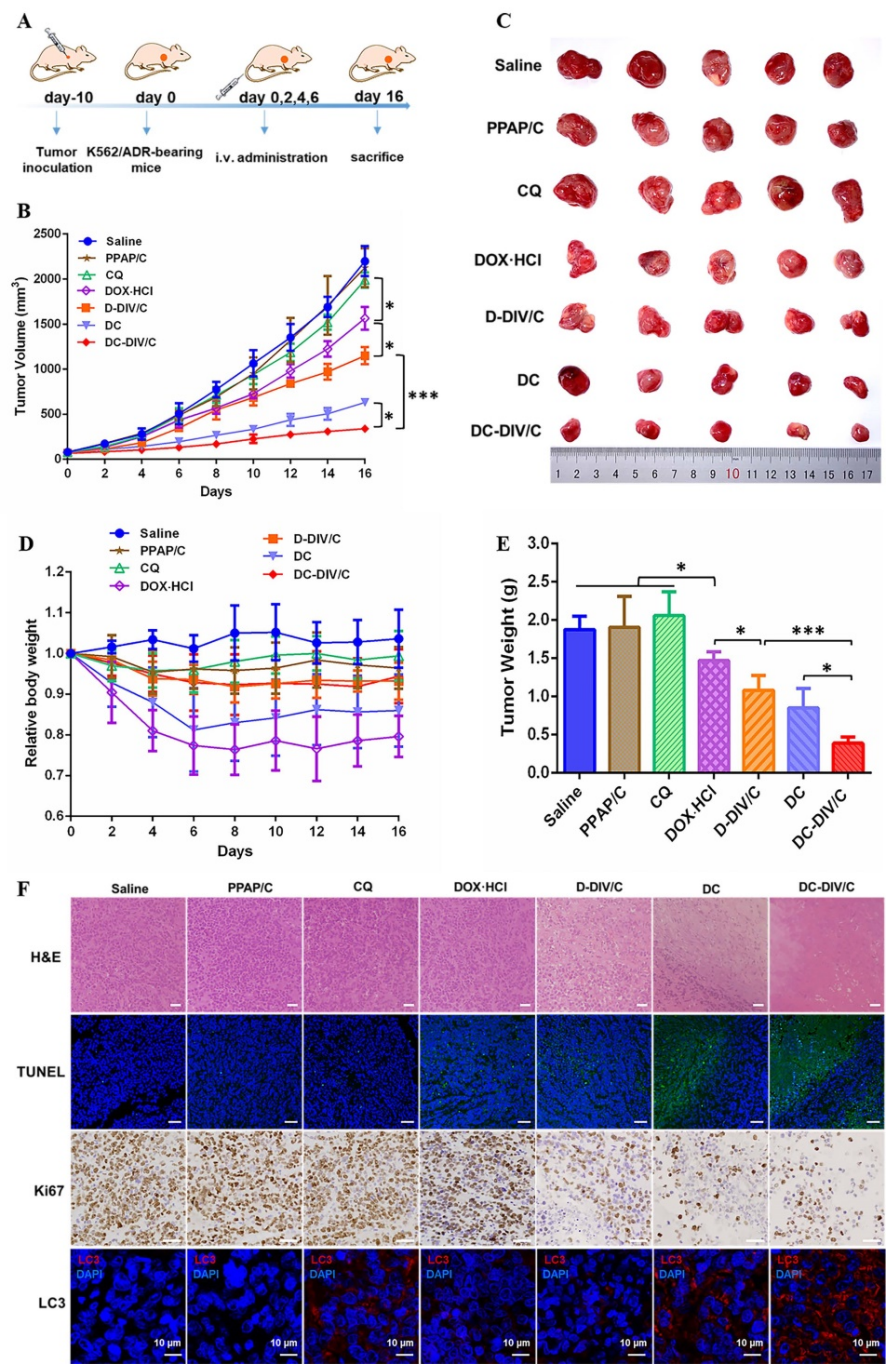
In vivo antitumor activity in K562/ADR-bearing nude mice. (A) Schedule of drug administration; (B) Changes in tumor volume of each group; (C) Tumor photographs on Day 16; (D) Body weights of mice after various treatments; (E) Tumor weight of each group on Day 16; (F) H&E, Ki67, TUNEL and LC3 analyses of tumor tissues after treatment (Scale bar = 50 μm and 10 μm). (* p < 0.05, *** p <0.001, n = 5, mean ± SD).

Inspired by the in vitro cytotoxicity results, we explored the in vivo anti-tumor efficacy of various formulations. The treatment schedule was shown in **Figure 7A**. The K562/ADR tumor-bearing mice were randomly divided into eight groups. Saline served as a control group, and PPAP/C was used to further study the toxicity of the carriers. The free drugs or drug-loaded nanovesicles were administered intravenously at 0, 2, 4, and 6 days. **Figure 7B** showed the growth curves of K562/ADR tumors in mice after treatment. For PPAP/C and free CQ, the tumor maintained rapid growth, showing very similar characteristics as those of the saline group. The therapeutic effect of free DOX·HCl on drug-resistant tumors was very limited, and the tumor inhibition rate (TIR) was only 28.80 %. Fortunately, adopting a co-delivering strategy for DOX⋅HCl and CQ, DC-DIV/C exhibited much stronger anti-tumor efficacy with the TIR of 84.52 %, displaying significant difference (*p<0.05) with free DC (TIR = 71.30%), and highly significant difference (***p <0.001) with D-DIV/C (TIR = 47.70 %).

To better evaluate the in vivo effects of various formulations, H&E staining, terminal deoxynucleotidyl transferase-mediated deoxyuridine triphosphate nick end labeling (TUNEL), and immunohistochemical (IHC) analyses (Ki67) were applied to characterize the status of tumor tissues (**Figure 7F**). H&E staining of tumor slices showed that the most obvious decreased cell density and much more necrosis for DC-DIV/C group compared with free drug and D-DIV/C, revealing a consistency with the above TIR values. Moreover, severe cell apoptosis (the green area) of DC-DIV/C was revealed by TUNEL assay and much less Ki67 positive cells presented by DC-DIV/C group were observed. These results validated again that DC-DIV/C could effectively suppress K562/ADR cell proliferation and induce more severe cell apoptosis. These results were consistent with the excellent TIR value presented by DC-DIV/C. To confirm the autophagy inhibition of CQ during the treatment, the endogenous LC3 protein in tumors was detected. The high expression of LC3Ⅱ (red spots) were shown in tumors treated with the formulations containing CQ, and LC3Ⅱ level was the highest in DC-DIV/C group, demonstrating a substantial accumulation of autophagosomes and the significant autophagy inhibition in tumor therapy.

The weight loss of various formulations was monitored during the treatment period. As shown in **Figure 7D**, both the free DOX·HCl and DC groups exhibited a sharp decrease in body weight and difficulty in recovery. Once DOX·HCl was encapsulated into the nanovesicles, however, this problem was effectively relieved, namely, D-DIV/C and DC-DIV/C groups showed minor body weight loss. Additionally, considering the cardiotoxicity of DOX·HCl, the histological studies of hearts were accomplished. As **Figure S9** shows, free DOX·HCl and DC groups exhibited severe histopathological abnormalities and lesions in the cardiac muscles. However, the similar histopathological abnormalities were not found after D-DIV/C or DC-DIV/C was administrated, indicating that DIV/C could effectively overcome the cardiotoxicity induced by DOX·HCl. Meanwhile, free CQ at the dosage of 10 mg/kg was safe for nude mice. Besides, no histopathological changes were found in the main organs such as liver, spleen, lung and kidney of various groups including DC-DIV/C.

In short, DC-DIV/C could effectively accumulate at tumor site, suppress tumor cell proliferation, induce massive apoptosis, and eventually cut down tumor volume significantly with the TIR of 84.52 %. These brilliant results could be ascribed to the following factors.

Firstly, the simultaneous encapsulation of DOX·HCl and CQ at the optimal ratio of 1:2 within nanovesicle was achieved on the basis of smart drug-driven nano-vesicle DIV/C, which was the precondition for enhancing in vivo anti-tumor action of DOX·HCl. Since DOX·HCl can complex with PPAP via hydrogen bond interaction and π-π conjugation but CQ cannot (**Figure S3A and S3B**), DOX·HCl with relatively low feeding content was able to induce the self-assemble of PPAP (**Figure 1Aa, a′**) and encapsulate CQ into the center aqueous cavity to form DC-DIV/C (**Figure 1Ab, b′**). The disparate location of the two water-soluble drugs within the same nano-vesicle presented an interesting phenomenon.

Secondly, drugs generally undergo diverse physiological fates upon systemic administration, especially for those with quite different pharmacokinetics behaviors. This is one major obstacle that separates success on in vitro cell models from actual in vivo outcomes. In this study, DC-DIV/C acted as an excellent vehicle for the synchronous delivery of DOX⋅HCl and CQ. Specifically, DC-DIV/C could inhibit the leakage of two drugs under physiological conditions (**Figure 1Bb**). Also as **Table 1** shows, the AUC of DOX⋅HCl and CQ in the free drug combination formulation (DC) was 1.03 μg·h/mL and 3.28 μg·h/mL, respectively, though the administration dosage ratio was 1:2. However, for DC-DIV/C, the AUC ratio of DOX⋅HCl to CQ was close to 1:2. Meanwhile, DC-DIV/C guaranteed the consistency of DOX·HCl and CQ in *t*_1/2β_ or MRT. These pharmacokinetic parameters suggested that DC-DIV/C owned ability to maintain DOX⋅HCl and CQ synchronously during blood circulation, assuring two drugs could reach tumor site at the optimal ratio. The similar rhythm at 1:2 (namely 0.5) was also occurred in cellular uptake of DC-DIV/C (**Figure 3C, D**), indicating that DOX·HCl and CQ could be synchronously captured by the cancer cells as the two drugs were co-loaded into DIV/C at the optimal ratio, whereas free DC could not. Later, due to the pH-responsive degradation of PPAP, DOX·HCl and CQ were quickly released from DC-DIV/C to exert their action against tumor (**Figure 1Bb**). Collectively, DC-DIV/C exhibited the synchronism character for DOX·HCl and CQ through the in vivo process of blood circulation, cellular uptake and intracellular release.

Finally, apart from the pharmacokinetic synchronism contributed by DIV/C, the autophagy blockade of CQ also plays a predominant role in MDR reversal. As a lysosomotropic agent, CQ could inhibit the lysosomal activity via increasing the pH in lysosomes, restrain degradation of the autophagic cargos and consequently block the fusion between autophagosomes and lysosomes as shown in** Figure 4B, C**. Moreover, the slight cell apoptosis triggered by CQ was observed (**Figure 2G-H**). Accordingly, due to a strong synergistic effect of DOX·HCl and CQ at 1:2 ratio (**Figure 2A-F**), the IC_50_ values of DOX·HCl produced by DC-DIV/C on MCF-7/ADR and K562/ADR cells decreased to 6.68 μg/mL and 2.21 μg/mL with significant reversal index of 10.80 and 8.56, indicating the powerful attack against MDR cells.

## Conclusions

With exquisite design, a drug-induced self-assembled nano-vesicle DC-DIV/C was constructed, which can co-deliver chemotherapeutic drug DOX⋅HCl and autophagy inhibitor CQ for significant MDR reversal and anti-tumor effect. Specially, the delivery synchronism of DOX⋅HCl and CQ during the in vivo process of blood circulation, cellular uptake and intracellular release was emphasized. This concept would be carried forward for more kinds of advanced combination therapy in clinics to achieve enhanced treatment outcomes.

## Supplementary Material

Supplementary methods, results, figures, and tables.

## Figures and Tables

**Table 1:** Pharmacokinetic parameters after intravenous administration of DC, D-DIV/C and DC-DIV/C at 5 mg/kg DOX·HCl or with 10 mg/kg CQ in rats (n = 3, mean ± SD).

Treatment	Drug measured	Pharmacokinetic parameters				
		*t*_1/2α_^a^ (h)	*t*_1/2β_^b^ (h)	AUC^c^ (μg·h/mL)	CL^d^ (L/h/kg)	MRT^e^ (h)
DC	DOX·HCl	0.03 ± 0.01	4.52 ± 0.58	1.03 ± 0.19	3.29 ± 0.48	4.17 ± 0.76
	CQ	0.22 ± 0.07	7.50 ± 1.51	3.28 ± 0.39	1.53 ± 0.22	8.03 ± 1.50
D-DIV/C	DOX·HCl	0.22 ± 0.09	37.72 ± 8.74	13.56 ± 0.49	0.27 ± 0.03	47.94 ± 12.81
DC-DIV/C	DOX·HCl	0.26 ± 0.17	33.70 ± 16.02	12.62 ± 2.12	0.28 ± 0.05	47.57 ± 22.68
	CQ	0.96 ± 0.50	35.89 ± 6.53	21.33 ± 0.66	0.19 ± 0.01	45.08 ± 6.88

^a^*t*1/2α: distribution half-life;^ b^*t*1/2β: elimination half-life; ^c^AUC: area under the plasma concentration-time curve; ^d^CL: total body clearance; ^e^MRT: mean residence time.

## Abbreviations

DOX·HCl: doxorubicin hydrochloride
CQ: chloroquine phosphate
DC: physical mixture of free DOX·HCl and CQ
PPAP/C: physical mixture of PPAP and cholesteryl hemisuccinate
DIV: drug-induced self-assembled nanovesicles
D-DIV/C: DOX·HCl loaded nanovesicles
DC-DIV: DOX·HCl and CQ co-loaded drug-induced self-assembled nanovesicles without cholesteryl hemisuccinate
DC-DIV/C: DOX·HCl and CQ co-loaded drug-induced self-assembled nanovesicles with cholesteryl hemisuccinate
RI: reversal index
CI: combination index
TIR: tumor inhibition rate
