# Supplementary material for: Drug-induced self-assembled nanovesicles for doxorubicin resistance reversal via autophagy inhibition and delivery synchronism
Source: Theranostics. 2022 May 13;12(8):3977–94. doi: 10.7150/thno.70852 (PMC9131275; doi:10.7150/thno.70852)
Supplement: Supplementary file 1 — Supplementary methods, results, figures, and tables. [file thnov12p3977s1.pdf]

Supplementary Material

Drug-induced self-assembled nanovesicles for doxorubicin resistance reversal via autophagy inhibition and delivery synchronism

Juan Wang, Liyan Qiu*

Ministry of Educational (MOE) Key Laboratory of Macromolecular Synthesis and Functionalization, Department of Polymer Science and Engineering, Zhejiang University, Hangzhou 310027, China

* Corresponding author: Prof. Liyan Qiu, Tel: +86 571 87952306, Fax: +86 571 87952306, E-mail: lyqiu@zju.edu.cn

This file includes:

Supplementary section 1. Methods.

Supplementary section 2. Results.

Supplementary Scheme S1 The synthesis routes of $mPEG_{2000}$-NH_2, ABD and PPAP.

Supplementary Figure S1 The ^1H NMR spectrum of ortho ester molecule (ABD).

Supplementary Figure S2 Characterization of PPAP.

Supplementary Figure S3 FT-IR characterization and TEM images of various formulations.

Supplementary Figure S4 The size measurements and TEM images of DC-DIV/C incubated in saline within 48 h.

Supplementary Figure S5 Cytotoxicity of different formulations against drug resistant and sensitive cells.

Supplementary Figure S6 Cytotoxicity results of PPAP/C against MCF-7/ADR, MCF-7, K562/ADR and K562 cells.

Supplementary Figure S7 Cellular uptake of DOX·HCl in MCF-7 and K562 cells.

Supplementary Figure S8 Investigation of P-gp expression and its functional activity.

Supplementary Figure S9 Hematoxylin and eosin (H&E) staining assay.

Supplementary Table S1 Particle size, drug loading content (LC) and encapsulation efficiency (EE) of various formulations.

Supplementary Table S2 Drug loading content (LC) and encapsulation efficiency (EE) of DC-DIV/C in saline for 24 h and 48 h.

Supplementary Table S3 The 50 % inhibitory concentration (IC_{50}) of DOX·HCl, CQ, D-DIV/C, DC-DIV/C against MCF-7 and K562 cells.

Supplementary section 1. Methods.

Synthesis of amphiphilic poly[(PEG)$_x$(ABD)$_y$phosphazene]$_n$ (PPAP)

The amphiphilic copolymer (PPAP) was synthesized by sequentially grafting NH_2-PEG_{2000} and ABD onto a poly(dichlorophosphazene) backbone through nucleophilic substitution (Scheme 1). Briefly, poly(dichlorophosphazene) was obtained by a ring-opening polymerization of hexachlorocyclotriphosphazene at 250 ℃. The obtained poly(dichlorophosphazene) (0.35 g, 3.02 mmol, -N=P-) was reacted with a certain amount of dehydrated $mPEG_{2000}$-NH_2 (0.88 g, 0.44 mmol) containing an equimolar amount of dry TEA in dry benzene solution for 24 h at 35 ℃. Then, an excess amount of ABD (0.84 g, 4.02 mmol) with equimolar amount of anhydrous TEA in dry benzene was added slowly into the above reaction system, and followed with another 48 h stirring at 50 ℃. All procedures were carried out under a dry nitrogen atmosphere. PPAP was obtained by precipitation in cold diethyl ether and then dialyzing against water, and freeze-dried finally.

The synthesis of ABD was carried out using three steps. Firstly, 3-amino-1,2-propanediol (15.00 g, 0.16 mol) was dissolved in anhydrous acetonitrile (50 mL), and then ethyl trifluoroacetate (21.66 mL, 0.18 mol) was added dropwise to the stirred solution under nitrogen protection at 0 ℃. Acetonitrile was removed by vacuum distillation after overnight reaction at room temperature and the residue was dissolved in ethyl acetate. Afterwards it was washed by 10 % $KHSO_4$ solution and saturated NaCl solution respectively. As a kind of colorless oil-like product, 2,2,2-trifluoro-*N*-(2,3-dihydroxypropyl) acetamide (Compound Ⅰ) was obtained as a kind of colorless oil-like

product by drying with anhydrous $MgSO_4$, filtration and vacuum concentration. Secondly, trimethyl orthoformate (25.00 mL, 0.22 mol) was added dropwise to the mixture of *p*-toluenesulfonic acid (0.1g, 0.5 mmol) and compound Ⅰ (10 g, 0.05 mol) in anhydrous acetonitrile under nitrogen protection. The reaction was stopped by adding 3 drops of trimethylamine after overnight reaction at room temperature. The product was dissolved in ethyl acetate after removing acetonitrile by vacuum distillation. Then it was washed by saturated Na_2CO_3 solution and saturated NaCl solution respectively. Analogously, 2,2,2-trifluoro-*N*-(2-methoxy-[1,3]-dioxolan-4-methylene) acetamide was acquired as a colorless oil-like product by the previous methods. Thirdly, compound Ⅱ (5.00 g, 21.83 mmol), pyridinium *p*-toluenesulfonate (0.06 g, 0.24 mmol) and benzyl alcohol (2.41 mL, 23.17 mmol) reacted for 4 h at 130 ℃ under nitrogen protection. After cooling to room temperature, the residue (Compound Ⅲ) was mixed with tetrahydrofuran (100 mL) and NaOH (2.0 M, 100 mL), and then stirred vigorously overnight. The residue was extracted with dichloromethane after removing tetrahydrofuran by vacuum distillation. As a yellow viscous liquid, 4-aminomethyl-2-benzyloxy-[1,3]- dioxolan (ABD, Compound Ⅳ) was obtained by drying the collected organic phase with anhydrous $MgSO_4$, filtering and vacuum concentration.

Cytotoxicity and annexin V-FITC/PI apoptosis assay

To investigate the cytotoxicity of free DOX·HCl, free CQ, physical mixture of free DOX·HCl and CQ, PPAP/C and drug-loaded nanovesicles, various cell lines (MCF-7, MCF-7/ADR, K562 and K562/ADR) were seeded in 96-well plates at $0.2\text{-}1.0\times10^4$ cells/well, respectively, and cultured in RPMI 1640 containing 10 % fetal bovine serum

at 37 °C with 5 % CO_2. After 24 h, cells were exposed to increasing concentrations of different formulations for 48 h. Then, 10 μL of CCK-8 reagent was added to each well and the plates were incubated for 2 h. And the absorbency was determined by a microplate reader (Thermo, USA) at 450 nm. Data are reported as means of triplicate measurements.

The apoptosis analysis was further investigated. Briefly, MCF-7/ADR or K562/MDR cells (2×10^5 cells/well) were seeded in 12-well plates and incubated overnight in RPMI 1640 containing 10 % fetal bovine serum. Afterwards, the cells were incubated with various formulations for 48 h at the final DOX·HCl and CQ concentration of 5 and 10 μg/mL, respectively. The cells were treated according to the kit instructions and analyzed by flow cytometry.

P-gp functional activity evaluation

Flow cytometry analysis was used to investigate the effect of CQ on the functional activity of P-gp through monitoring the intracellular uptake of rhodamine 123, namely, a kind of P-gp substrate. Specifically, MCF-7/ADR cells and K562/ADR cells (2×10^5 cells/well) were seeded in 12-well plates, respectively. 24 h later, the cells were incubated in the medium containing various concentrations of free CQ for a certain period. Afterwards, rhodamine 123 (2 μg/mL) was incubated with these cells for 30 minutes at 37°C. Finally, the cells were washed with precooling PBS (pH 7.4) three-times, collected, and resuspended in PBS. Then the cell suspension was analyzed by flow cytometry with the 488 nm argon ion laser. Also, the individual fluorescence of 10^4 cells was collected for each sample at the appropriate period.

Western blotting

The expression of P-gp, autophagy related marker protein LC3 and autophagy substrate protein p62 was analyzed by western blot. MCF-7/ADR cells and K562/ADR cells were washed three times using ice-cold PBS (pH7.4), collected and lysed using NP40 lysis buffer (Beyotime, China) after incubation of various formulations with DOX·HCl concentration of 15 μg/mL and CQ concentration of 30 μg/mL for 48 h. The protein concentration was determined by BCA protein assay and equal micrograms of proteins were electrophoresed by SDS-polyacrylamide gel electrophoresis (SDS-PAGE). Then the proteins were transferred to polyvinylidene difluoride membrane (PVDF, Millipore) under ice conditions. Membranes were incubated with the corresponding primary antibodies overnight at 4 °C after blocked with 5 % fat-free milk. Then membranes were washed and incubated with the corresponding secondary antibody for 1 h at room temperature. The membranes were visualized on the Odyssey® scanner (LI-COR Biosciences, Lincoln, NE). In addition, the expression of P-gp in MCF-7 cells and K562 cells was also investigated with the same protocol.

Investigation of cellular autophagy by bio-TEM

MCF-7/ADR cells and K562/ADR cells were cultured in culture flasks at a density of 2×10^6 cells for 24 h. Then, PBS and DC-DIV/C with DOX·HCl concentration of 5 μg/mL and CQ concentration of 10 μg/mL were added for 24 h, respectively. After that, the cells were washed twice with precooling PBS and collected. These collected cells were fixed with 2.5 % glutaraldehyde solution for 12 h at 4 °C and then post-fixed with 1 % osmium tetroxide for 1 h at room temperature. These fixed cells were dehydrated

with ethanol and embedded. The samples were obtained by ultrathin section technique and staining. These final samples were observed by the HT-7700 TEM (Hitachi, Tokyo, Japan).

Pharmacokinetic study

In brief, DOX·HCl in the plasma samples was extracted with the equivalent volume of acetonitrile after vigorously vortexing for 5 min, and then centrifuged at 10 000 rpm for 5 min, the supernatant was injected into the HPLC system. A Venusil XBP C18 reverse-phase column (4.6 mm i.d. × 250 mm) was used, and the mobile phase consisted of acetate buffer (pH 5.0)/methanol/ acetonitrile (50/25/25, v/v/v). The flow rate was maintained at 0.8 mL/min, and the UV detection wavelength was set as 254 nm. CQ in the plasma samples was extracted with the equivalent volume of isometric methanol-water (v/v, 1:1) as the previous operation. The supernatant was injected into the HPLC system using the same column, and the mobile phase consisted of 1.4 g/L dibasic sodium phosphate buffer (pH 3.0)/methanol (30/70, v/v). The flow rate was maintained at 0.8 mL/min, and the UV detection wavelength was set as 260 nm.

Supplementary section 2. Results.

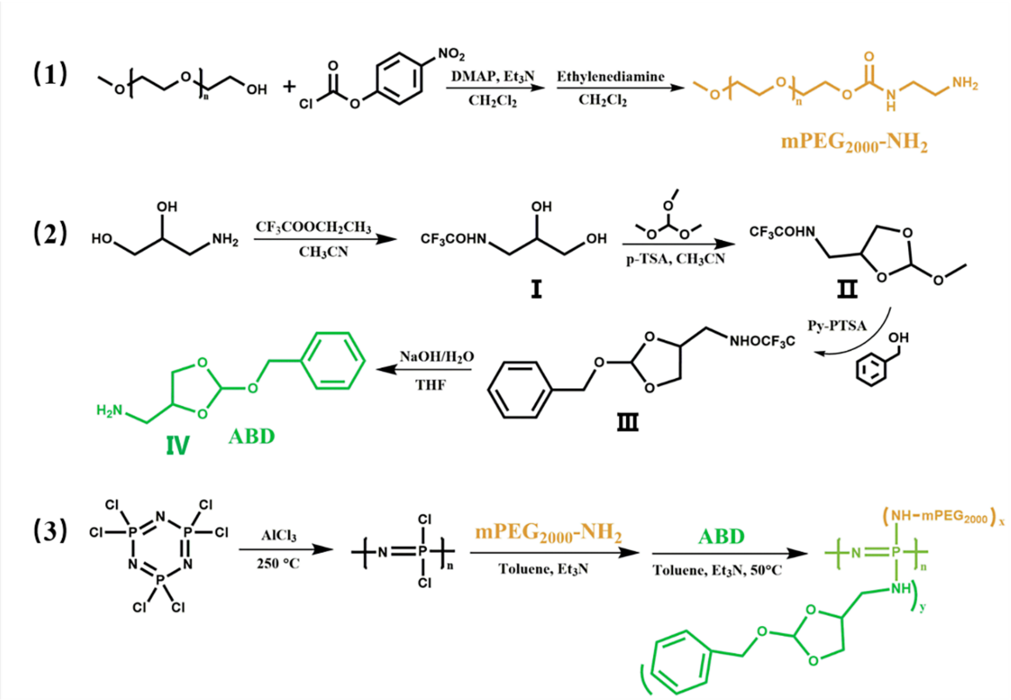

Scheme S1. The synthesis routes of $mPEG_{2000}$-NH_2, ABD and PPAP.

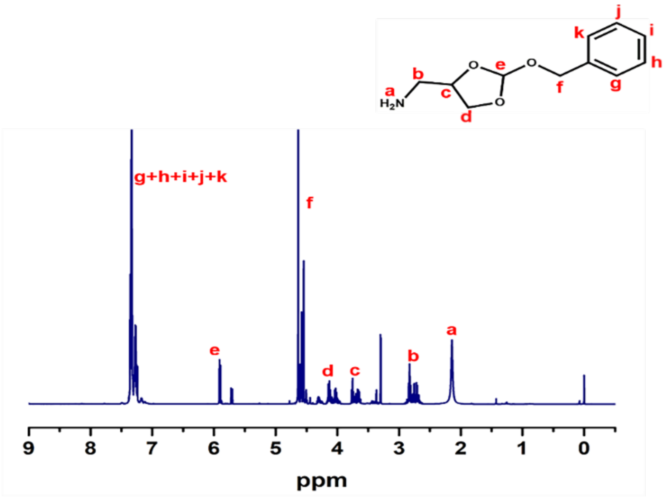

Figure S1. The ^{1}H NMR spectrum of ortho ester molecule (ABD).

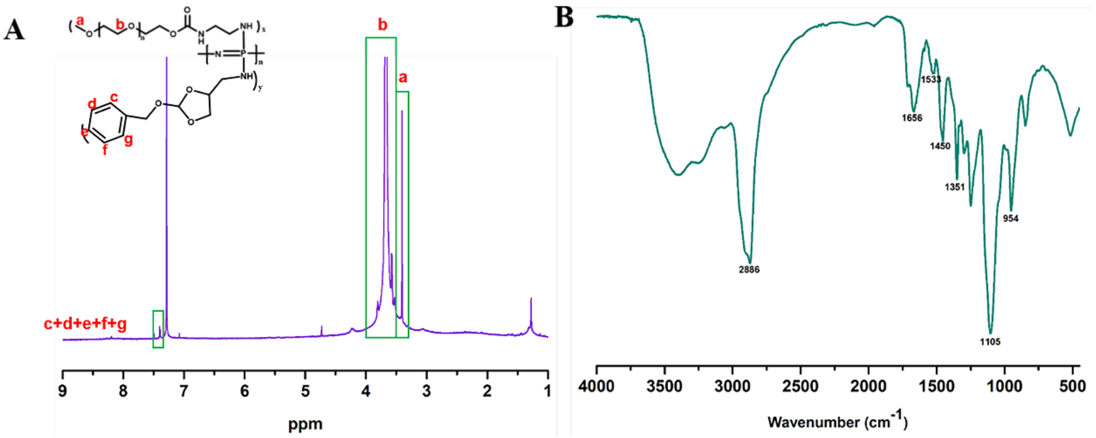

Figure S2. Characterization of PPAP. **(A)** ^{1}H NMR spectrum of PPAP; **(B)** FT-IR spectrum of PPAP.

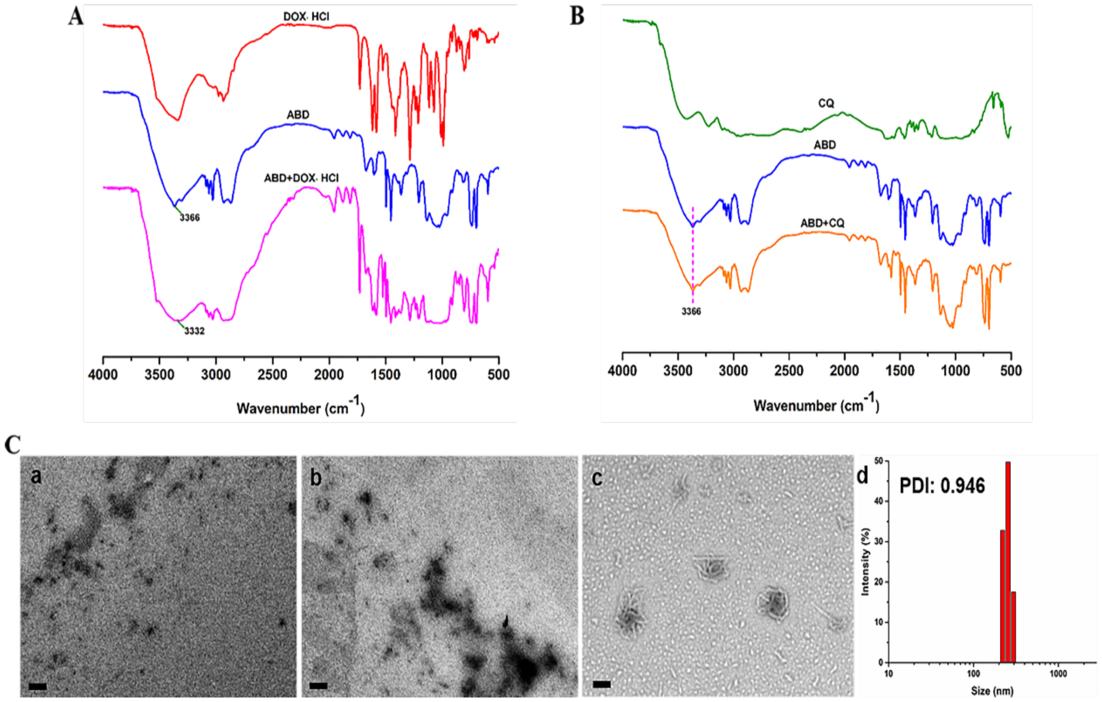

Figure S3 FT-IR characterization and TEM images of various formulations. **(A)** FT-IR spectra of DOX·HCl, ABD and mixture of ABD and DOX·HCl; **(B)** FT-IR spectrum of CQ, ABD and mixture of ABD and CQ; **(C)** TEM images of (a) PPAP/C; (b) mixture of CQ and PPAP/C; (c) DC-DIV/C incubated in pH 5.5 phosphate buffer for 1 h; (d) DLS result of DC-DIV/C incubated in pH 5.5 phosphate buffer for 1 h. Scale bars = 0.2 μm.

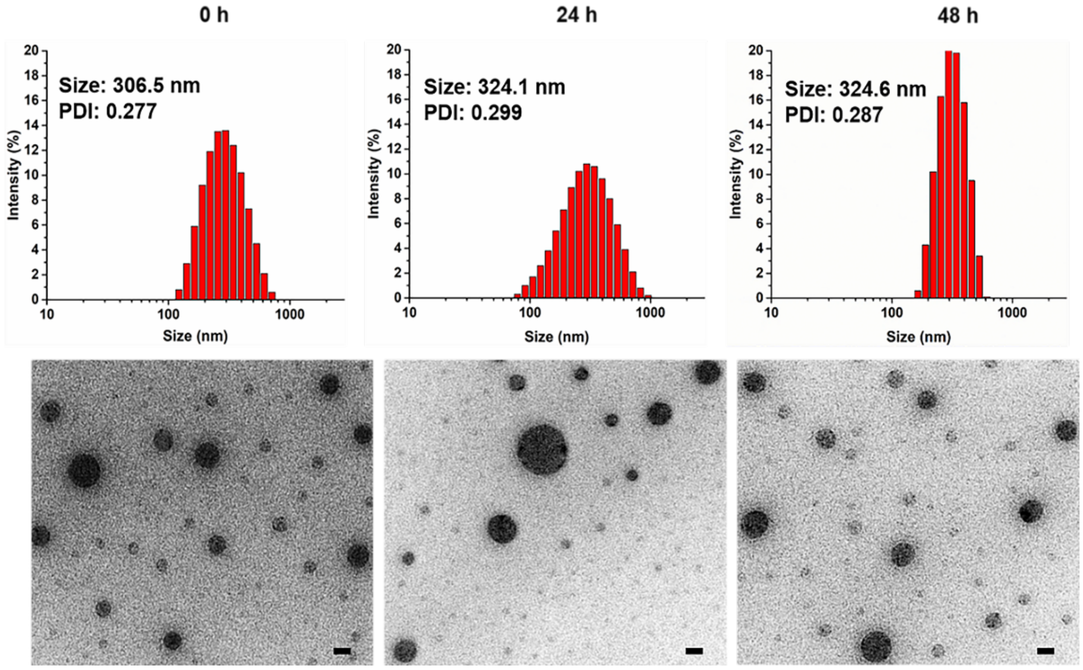

Figure S4 The size measurement by DLS and TEM images of DC-DIV/C incubated in saline within 48 h. Scale bars = 0.2 μm.

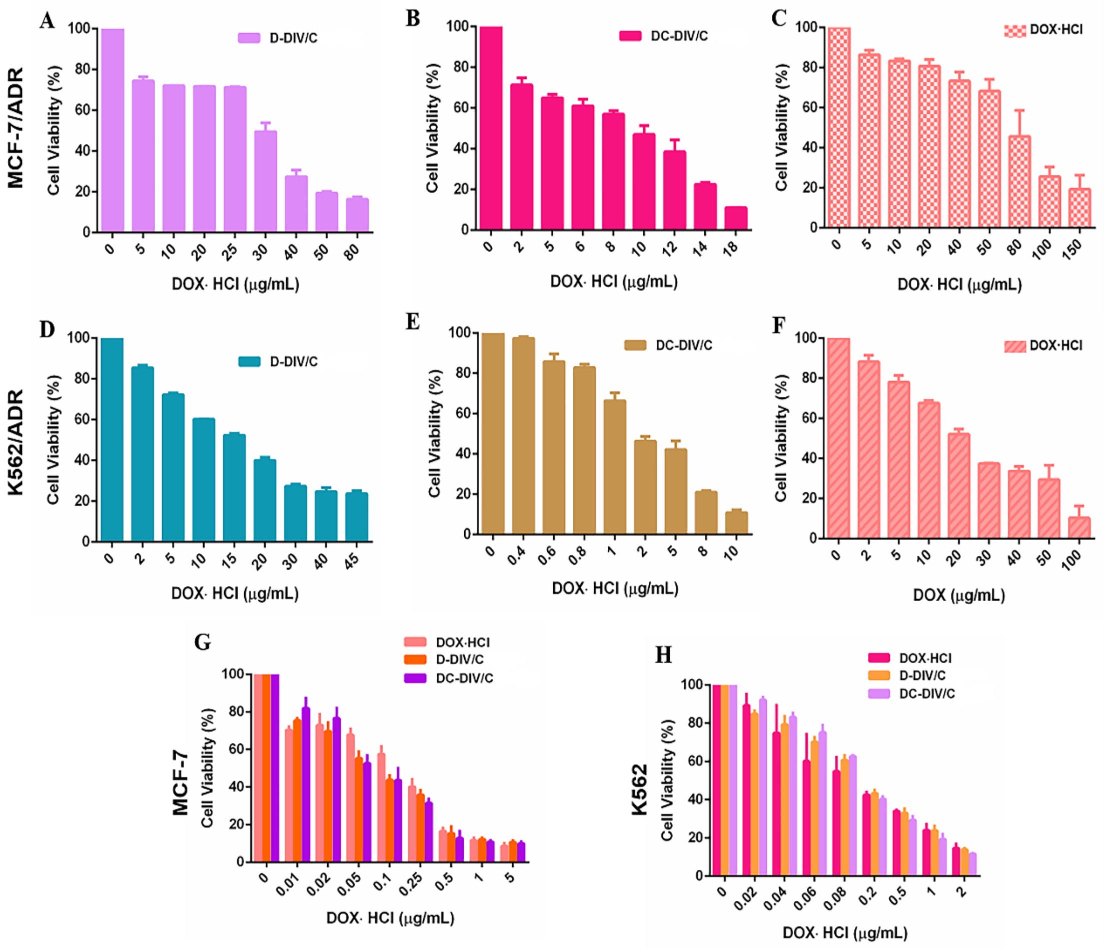

Figure S5 Cytotoxicity of different formulations against drug resistant and sensitive cells. (A) Cytotoxicity of D-DIV/C against MCF-7/ADR cells (48 h); (B) Cytotoxicity of DC-DIV/C against MCF-7/ADR cells (48 h); (C) Cytotoxicity of DOX·HCl against MCF-7/ADR cells (48 h); (D) Cytotoxicity of D-DIV/C against K562/ADR cells (48 h); (E) Cytotoxicity of DC-DIV/C against K562/ADR cells (48 h); (F) Cytotoxicity of DOX·HCl against K562/ADR cells (48 h); (G) Cytotoxicity of DOX·HCl, D-DIV/C and DC-DIV/C against MCF-7 cells (48 h); (H) Cytotoxicity of DOX·HCl, D-DIV/C and DC-DIV/C against K562 cells (48 h). (mean ± SD, n = 3).

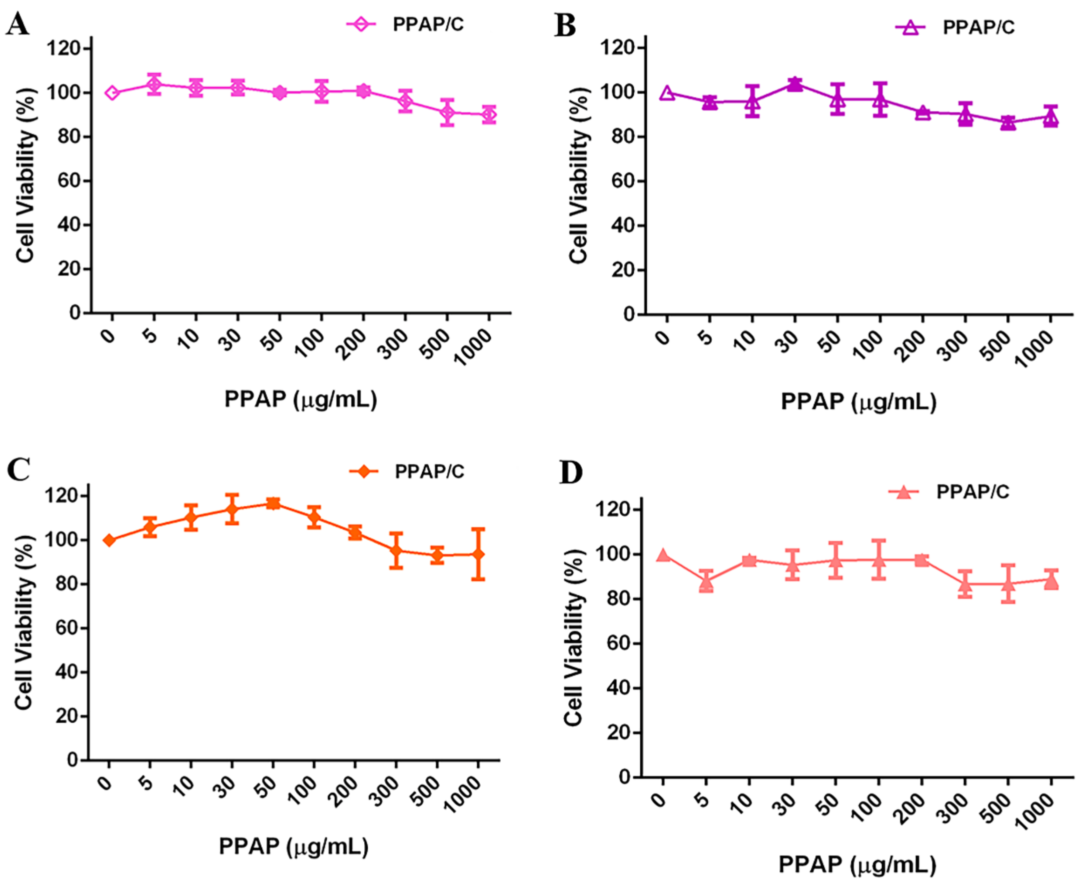

Figure S6 Cytotoxicity results of PPAP/C against various cells. (A) MCF-7/ADR cells, (B) MCF-7 cells, (C) K562/ADR cells and (D) K562 cells within 48 h.

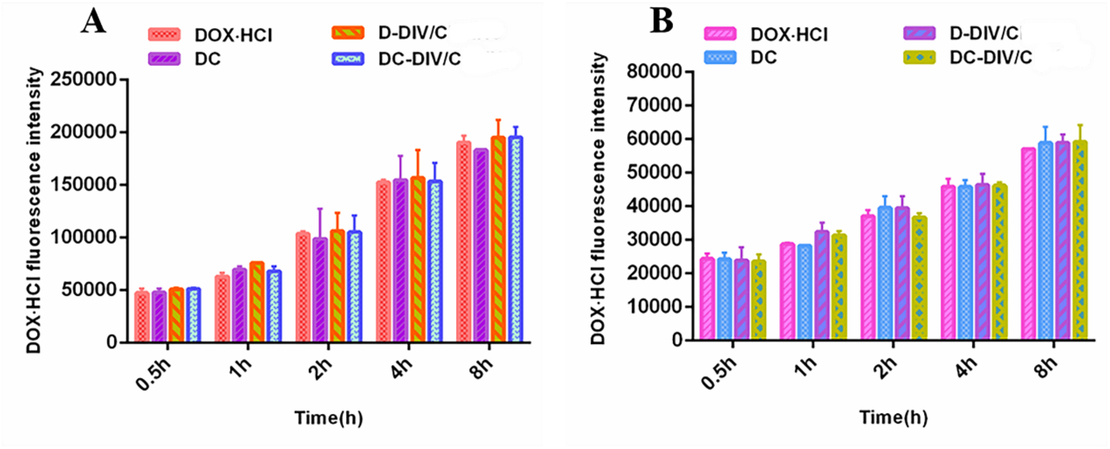

Figure S7 Cellular uptake of DOX·HCl of various formulations in (A) MCF-7 cells and (B) K562 cells. (mean ± SD, n = 3).

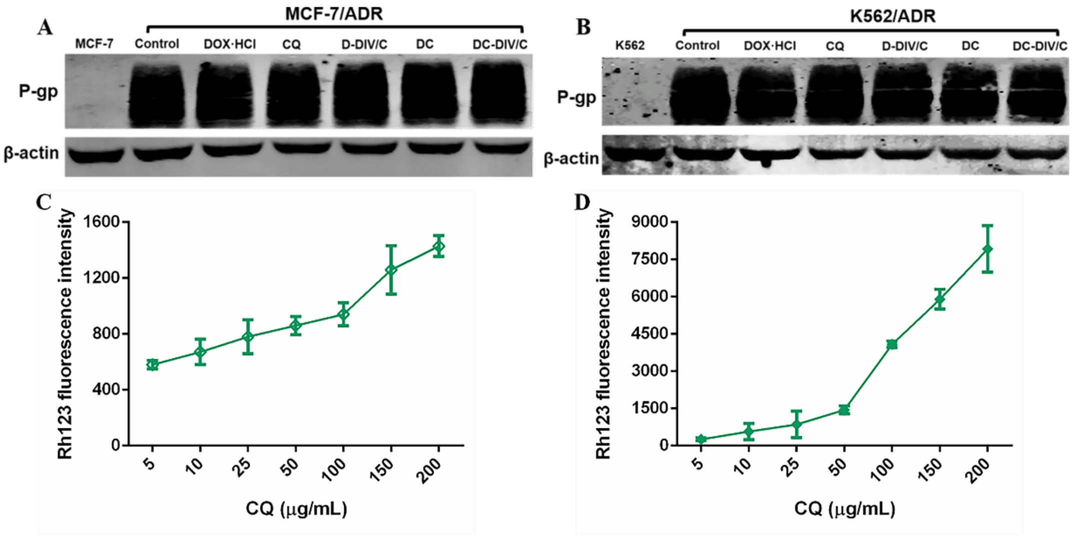

Figure S8 Investigation of P-gp expression and its efflux activity. Western blot analysis of P-gp protein expression in (A) MCF-7 cells, MCF-7/ADR cells and (B) K562 cells, K562/ADR cells; Cellular accumulation of rhodamine 123 in (C) MCF-7/ADR cells and (D) K562/ADR cells. (mean ± SD, n = 3).

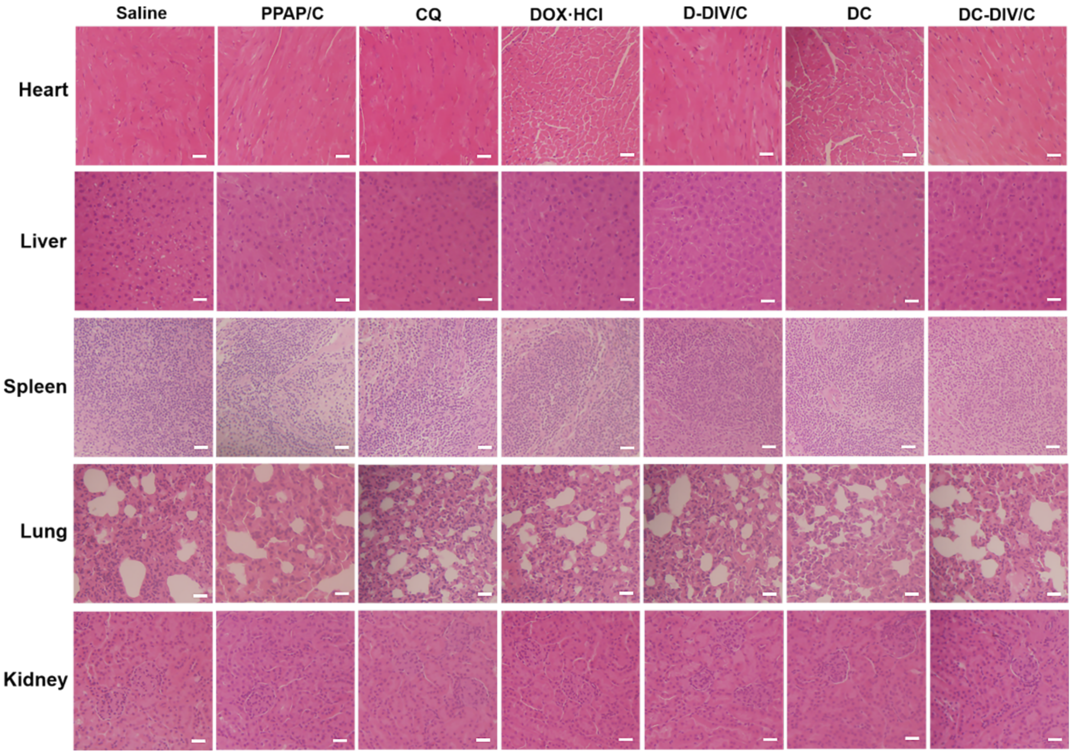

Figure S9 Hematoxylin and eosin (H&E) staining assay of the hearts, livers, spleens, lungs and kidneys collected from different groups of mice at the end of treatments (day 16). Scale bars: 50 µm.

Table S1 Particle size, drug loading content (LC) and encapsulation efficiency (EE) of various formulations. (mean ± SD, n = 3).

Sample	Theoretical LC % of DOX·HCl	Theoretical LC % of CQ	Size (nm) (PDI)	LC (%)		EE (%)	
				DOX·HCl	CQ	DOX·HCl	CQ
DIV	7.0	/	163.1 ± 0.3 (0.2)	4.6 ± 0.1	/	68.9 ± 1.2	/
D-DIV/C	20.0	/	302.2 ± 25.4 (0.3)	13.2 ± 0.4	/	72.8 ± 8.0	/
DC-DIV	7.0	23.0	177.1± 11.3 (0.2)	4.3 ± 0.2	8.7 ± 1.8	72.7 ± 3.0	43.7 ± 8.8
DC-DIV/C	7.0	23.0	327.4 ± 9.5 (0.2)	4.6 ± 0.3	8.7 ± 0.5	77.4 ± 5.8	43.3 ± 2.6

Table S2 Drug loading content (LC) and encapsulation efficiency (EE) of DC-DIV/C after incubated in saline for 24 h and 48 h. (mean ± SD, n = 3).

**Time (h)**	**LC (%)**		**EE (%)**	
	**DOX·HCl**	**CQ**	**DOX·HCl**	**CQ**
0	4.4 ± 0.4	8.6 ± 0.3	75.2 ± 6.9	43.1 ± 1.5
24	4.4 ± 0.2	8.6 ± 0.2	74.0 ± 2.9	42.8 ± 1.0
48	4.3 ± 0.1	8.6 ± 0.6	72.3 ± 1.7	42.9 ± 2.9

Table S3 The 50 % inhibitory concentration (IC_{50}) of DOX·HCl, CQ, D-DIV/C, DC-DIV/C against MCF-7 cells and K562 cells within 48 h. (mean ± SD, n = 3).

**Treatment**	**MCF-7**	**K562**
	**IC_{50} (μg/mL)**	**IC_{50} (μg/mL)**
CQ	23.85 ± 0.42	17.44 ± 1.34
DOX·HCl	0.08 ± 0.01	0.15 ± 0.06
D-DIV/C	0.06 ± 0.01	0.17 ± 0.01
DC-DIV/C	0.08 ± 0.01	0.18 ± 0.02
